# Divergent splicing factor SRSF1 signaling promotes inflammation post-CME: the SRSF1/ENPP3 axis acts via inhibition of BRD4 O-GlcNAcylation to enhance NF-κB activation and accelerate heart failure

**DOI:** 10.7150/thno.115402

**Published:** 2025-06-09

**Authors:** Chen-Kai Hu, Lei He, Wan-Zhong Huang, Yuan Huang, Ri-Xin Dai, Chen Chang, Jun-Xiong Qiu, Qiang Wu, Qiang Su

**Affiliations:** 1 Department of Cardiology, the Second Affiliated Hospital, Jiangxi Medical College, Nanchang University, No. 1 Minde Road, Nanchang, Jiangxi, 330006, China.; 2 Department of Cardiology, Jiangbin Hospital of Guangxi Zhuang Autonomous Region, No. 85 Hedi Road, Nanning, Guangxi, 530021, China.; 3 Department of Cardiology, the First Affiliated Hospital of Guilin Medical University, Guilin, Guangxi, 541001, China.; 4 Department of Cardiovascular Surgery, Sun Yat-sen Memorial Hospital, Sun Yat-sen University, Guangzhou, 510120, China.; 5 Senior Department of Cardiology, the Sixth Medical Center, Chinese PLA General Hospital, Beijing, 100048, China.

**Keywords:** coronary microembolization, inflammation, SRSF1, ENPP3, BRD4

## Abstract

**Rationale:** Coronary microembolization (CME) is a severe medical condition that occurs during acute coronary syndrome, leading to myocardial inflammation, apoptosis, and cardiac dysfunction. The research investigated SRSF1 biological functions during myocardial inflammation caused by CME and its underlying mechanisms.

**Methods:** CME models were established in rats injected with microspheres in the left ventricle and oxygen-glucose deprivation (OGD)-exposed cardiomyocytes. RT-qPCR, Western blotting and immunohistochemical staining were used to evaluate the expression of target molecules. Myocardial apoptosis was detected by flow cytometry. The direct binding between SRSF1 and ectonucleotide pyrophosphatase/phosphodiesterase 3 (ENPP3) was verified by RIP and TRAP. Protein interaction was determined by Co-IP. The dual-luciferase reporter assay measured inflammatory cytokine transcription levels. Myocardial injury was assessed by HE staining and ultrasound examinations. The study used ELISA to measure inflammatory cytokines and cardiac troponin I (cTnI) levels.

**Results:** SRSF1 expression was strikingly enhanced in CME models. Knockdown of SRSF1 effectively restrained NF-κB-mediated myocardial inflammation through increasing ENPP3 mRNA/lncRNA ENPP3 ratio by regulating alternative splicing of ENPP3 pre-mRNA. The GlcNAcylation of bromodomain-containing protein 4 (BRD4) was reduced during CME, which increased BRD4 protein level to trigger NF-κB-mediated inflammation. SRSF1/ENPP3 axis inhibited the GlcNAcylation of BRD4 in CME. Myocardial-specific knockout of SRSF1 restored cardiac function and restrained myocardial inflammation in CME rats by inactivation of the ENPP3/BRD4/NF-κB pathway.

**Conclusions:** SRSF1 facilitates CME-induced myocardial inflammation by up-regulating ENPP3/lncRNA ENPP3 ratio to suppress GlcNAcylation of BRD4, suggesting SRSF1 inhibition as a promising therapeutic strategy for CME.

## Introduction

Coronary microembolization (CME) is a severe complication that can arise from percutaneous coronary intervention, affecting 15% to 20% of patients 1, 2. CME is characterized by occlusion of coronary micro-vessels induced by tiny thrombi or plaque debris 3. CME can result in slow/no-reflow flow in myocardia, which may consequently cause myocardial inflammation and necrosis/apoptosis 4. It has been recognized that CME-induced inflammation is a key driver of myocardial dysfunction 5. Therefore, inhibition of myocardial inflammation is considered as an effective strategy for ameliorating CME-induced myocardial dysfunction. However, the pathogenesis of CME-induced myocardial inflammation remains largely unknown.

The SR protein family includes Serine-arginine-rich splicing factor 1 (SRSF1) as one of its essential members. SRSF1 has been identified to be a modulator of alternative splicing via direct interaction with exonic splicing enhancer sequences 6. For instance, SRSF1 functioned as a *trans-*acting factor to inhibit CD33 exon 2 skipping, thereby enhancing the expression of full-length CD33 transcript 7. SRSF1 plays a role in the development of myocardial inflammation according to prior findings. Lin *et al.* documented that circDiaph3 exacerbated myocardial inflammation via modulation of miR-338-3p/SRSF1 axis 8. Another study indicated that SRSF1 participated in lncRNA MAAMT-induced myocardial inflammation by promoting macrophage activation 9. Our initial research showed that SRSF1 expression levels increased in rat myocardial tissues that experienced ischemia. Therefore, we speculated that SRSF1 might participate in myocardial inflammation during CME.

Ectonucleotide pyrophosphatase/phosphodiesterase 3 (ENPP3) belongs to ENPP family, which takes part in the regulation of glycosyltransferase 10. There are two transcripts of ENPP3 gene via alternative splicing: one is ENPP3 mRNA transcript containing 27 exons; the other is lncRNA ENPP3 transcript with exon 21 skipping. We used starBase and RBPDB databases to predict the potential RNA-binding protein (RBP) of ENPP3. Interestingly, by overlapping the results, SRSF1 was predicted to bind to ENPP3. Nevertheless, whether alternative splicing of ENPP3 can be modulated by SRSF1 in CME-induced inflammation remains obscure.

Proteins undergo O-GlcNAcylation modification through the attachment of N-acetylglucosamine (GlcNAc) to their targets. O-GlcNAcylation functions as a reversible modification which controls various pathophysiological processes including inflammation. For example, O-GlcNAc affected innate immune cell function via regulation of proteins in inflammation-related pathways 11. Silva *et al.* documented that O-GlcNAcylation could alleviate LPS-induced systemic inflammation in mice 12. Notably, a previous study found that ENPP3 could hydrolyze UDP-GlcNAc, the only donor substrate for O-GlcNAc, suggesting that ENPP3 might be a negative regulator of O-GlcNAcylation 10. So far, the influence of SRSF1/ENPP3 axis on O-GlcNAcylation during CME-induced inflammation has not been elucidated.

Bromodomain-containing protein 4 (BRD4) has been identified as a coactivator of NF-κB via interaction with the acetylated lysine-310 of NF-κB, which can facilitate the initiation and development of inflammation 13. Besides, BRD4 could interact with NF-κB at the super-enhancers of its target genes, thereby facilitating the transcription of pro-inflammatory cytokines 13. Dong *et al.* reported that O-GlcNAcylation of NF-κB p65 could restrain inflammation and astrocyte activation in mice with Alzheimer's disease 14. However, whether BRD4 can be modulated by O-GlcNAcylation during cardiomyocyte inflammation in CME remains unknown. Thus, we demonstrated that up-regulation of SRSF1 repressed exon 21 skipping of ENPP3 pre-mRNA to enhance ENPP3 protein level, which inhibited O-GlcNAcylation of BRD4 to activate NF-κB pathway, thus aggravating CME-induced cardiomyocyte inflammation. Our findings uncover the modulatory roles and related mechanisms of SRSF1 in CME-induced inflammation.

## Results

### SRSF1 was highly expressed in the* in vivo* and *in vitro* CME models

**Figure 1:**
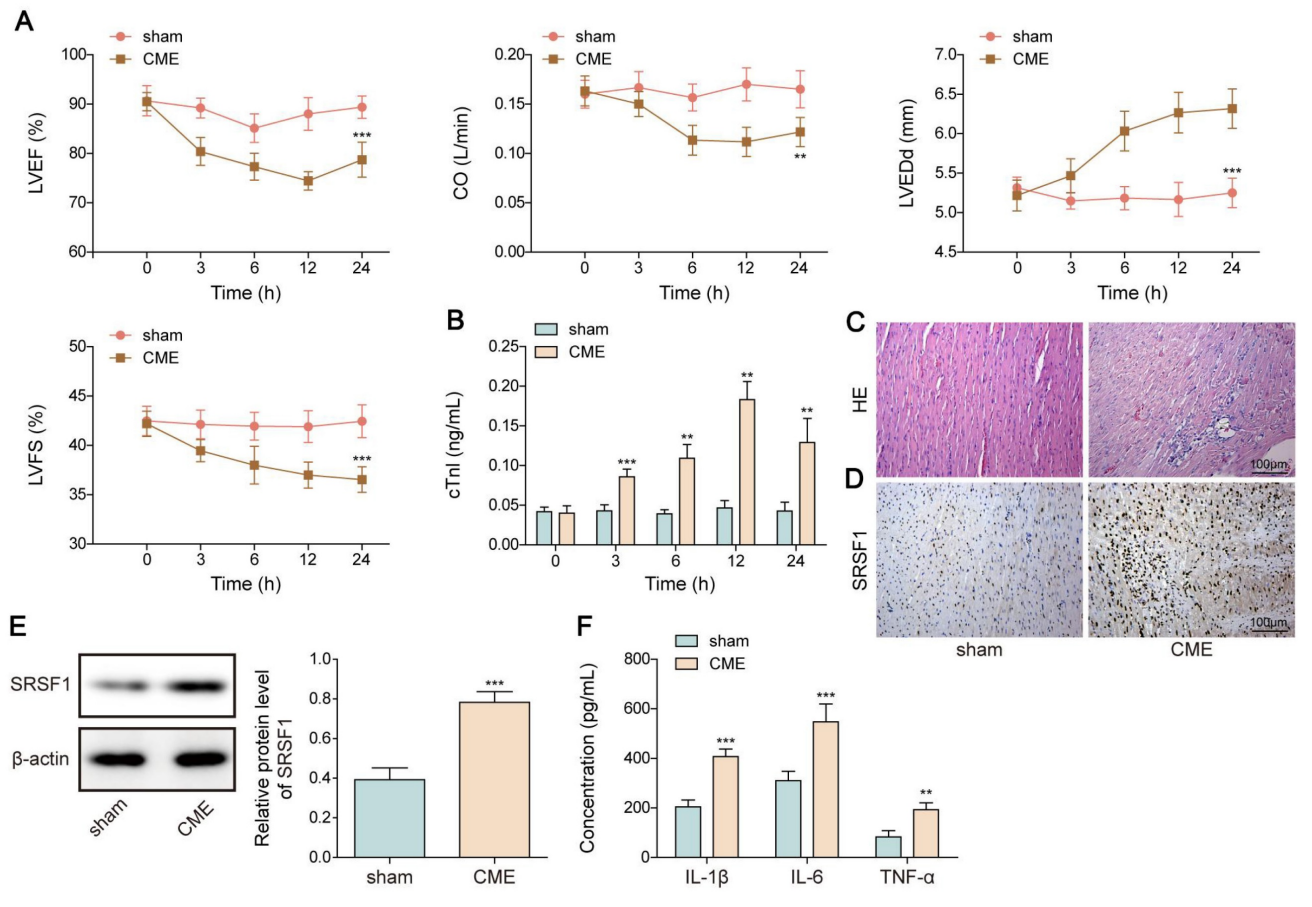

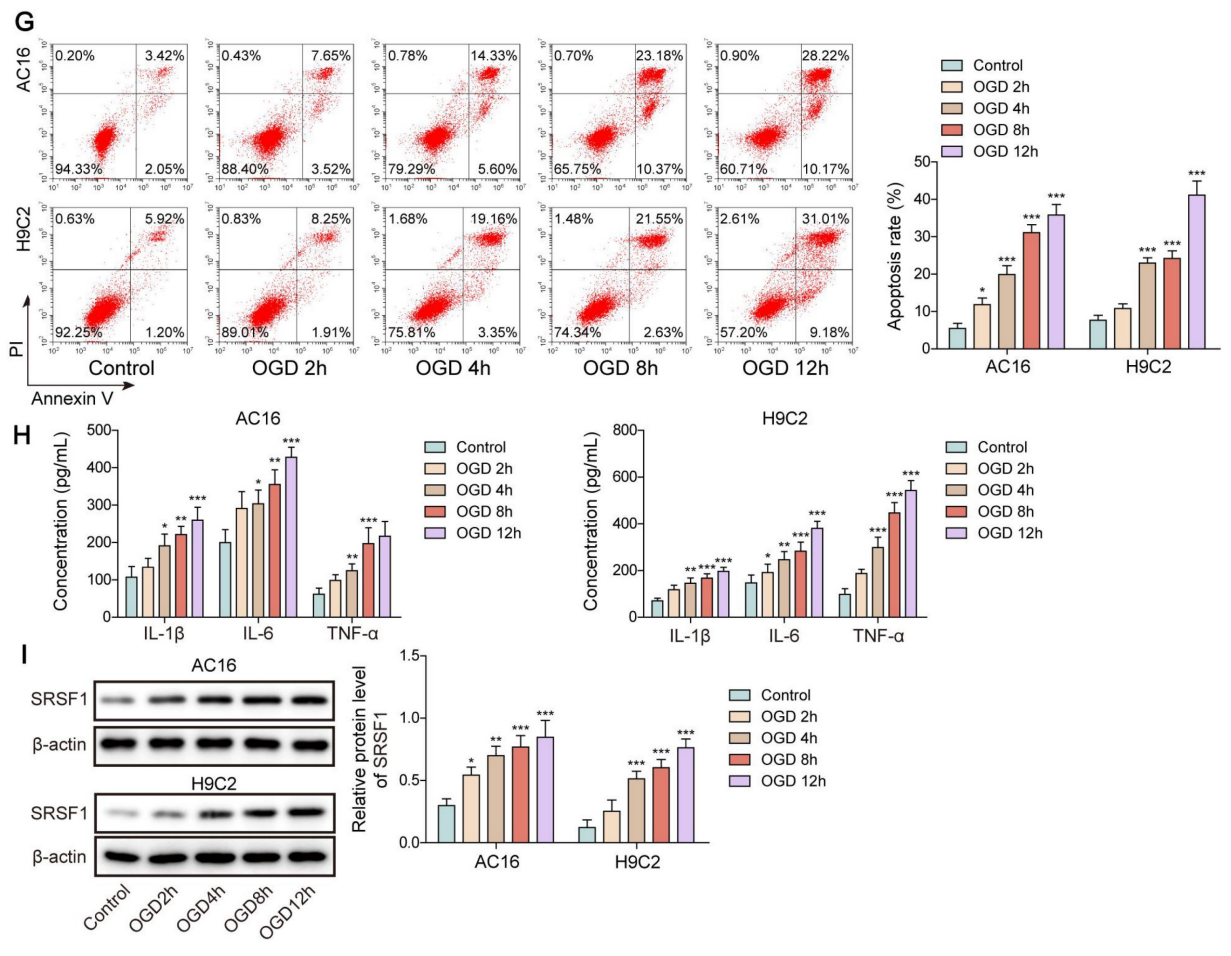
Aberrant high expression of SRSF1 in CME models. CME model was established in rats by injection of microspheres into the left ventricle. (A) LVEF, LVFS, LVEDd, and CO in each group were determined. (B) The serum cTnI level was assessed by ELISA. (C) Myocardial tissues were subjected to HE staining (scale bar = 100 μm). (D) SRSF1 expression in myocardial tissues was detected by immunohistochemical staining (scale bar =100 μm). (E) Western blotting analysis of SRSF1 expression in myocardial tissues. (F) The serum TNF-α, IL-1β, and IL-6 levels were measured by ELISA. H9C2 and AC-16 cells were stimulated with OGD for 2, 4, 8, 12 h to mimic CME* in vitro*. (G) Apoptotic percentage was evaluated by flow cytometry. (H) The release of TNF-α, IL-1β, and IL-6 from cardiomyocytes was determined by ELISA. (I) Western blotting analysis of SRSF1 level in cardiomyocytes. For A-F, n=6. For G-I, n=3. ANOVA for repeated measurement (for A, B), Student's t test (for E, F) and one-way ANOVA (for G-I) were performed to analyze data. *p < 0.05, **p < 0.01, ***p < 0.001.

First, a rat model of CME was used to validate the SRSF1 dysregulation. We observed cardiac dysfunction in CME rats as evidenced by reduction in LVEF, LVFS, and CO levels, but elevation in LVEDd level (**Figure 1A**). Besides, serum cTnI level was time dependently increased within 12 h of CME, which was subsequently declined slightly (**Figure 1B**). Obvious lesions were found in the myocardium of CME rats, such as inflammatory cell infiltration, edema, and microembolism (**Figure 1C**). The expression of SRSF1 in the hearts of CME rats was higher as compared with sham rats (**Figure 1D, E**). Notably, the inflammatory cytokines TNF-α and IL-1β and IL-6 were released by CME (**Figure 1F**). Furthermore, an *in vitro* model of CME was constructed in OGD-challenged cardiomyocytes. The exposure of cardiomyocytes to OGD resulted in time-dependent increases of apoptosis and pro-inflammatory cytokine production (**Figure 1G, H**). The *in vitro* findings were confirmed by the increased SRSF1 expression that occurred with longer OGD stimulation (**Figure 1I**). Therefore, SRSF1 expression was abnormally elevated during CME progression.

### SRSF1 directly bound to ENPP3 pre-mRNA to control its alternative splicing

**Figure 2:**
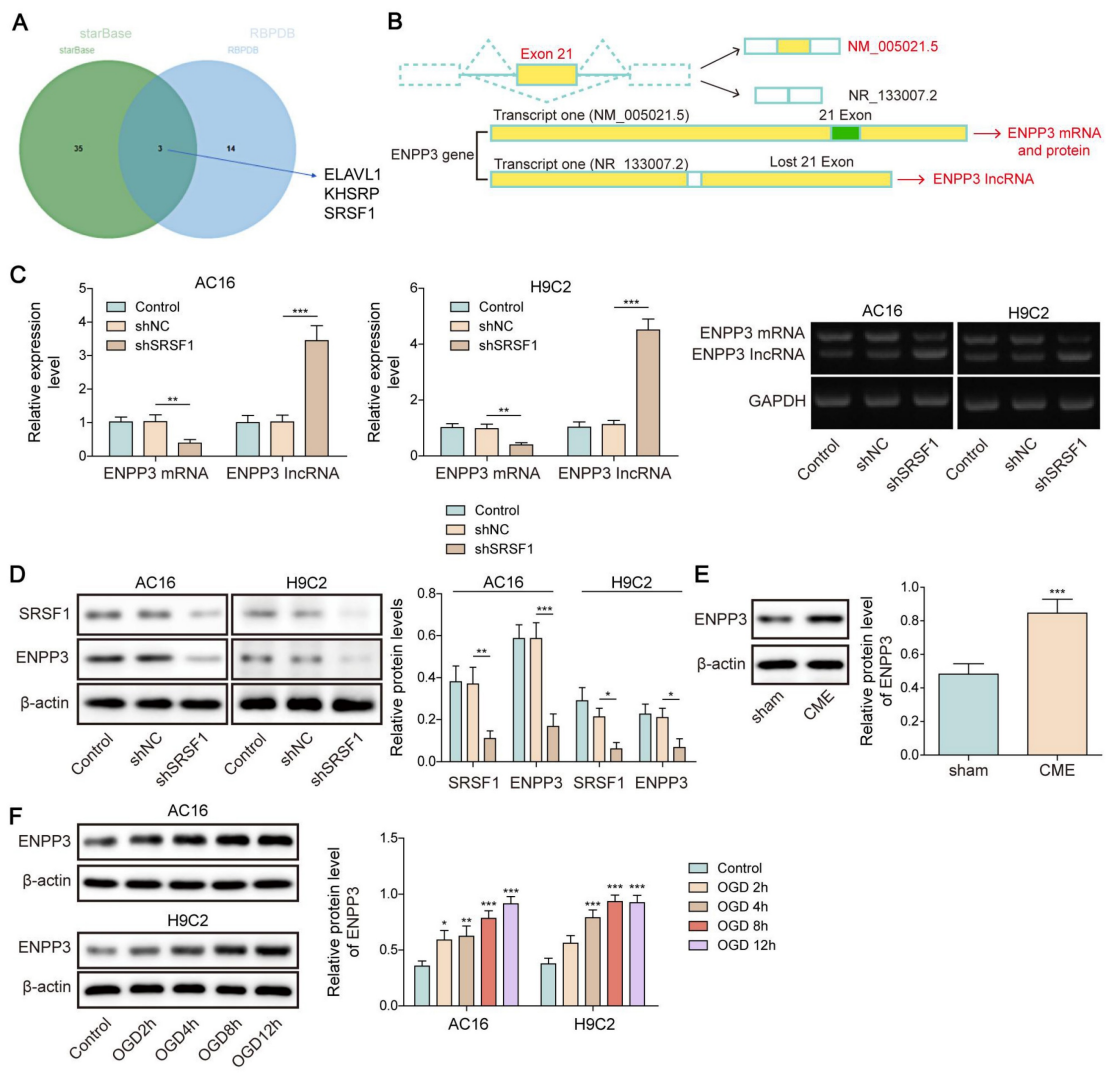

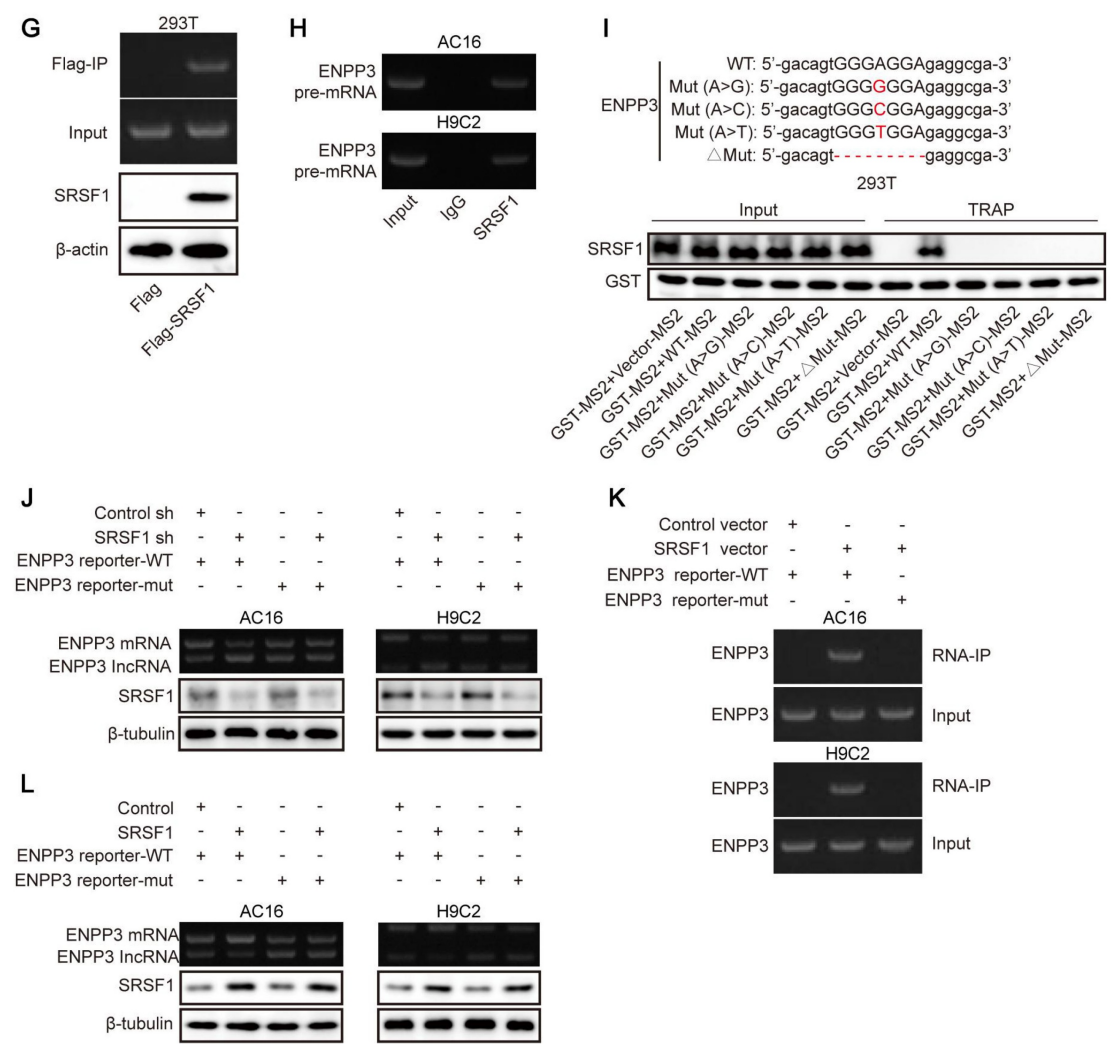
SRSF1 regulated alternative splicing of ENPP3 pre-mRNA. (A) StarBase and RBPDB databases were used to predict the potential RNA-binding protein (RBP) of ENPP3. (B) Illustration of two transcripts of ENPP3 gene and the potential binding sites of SRSF1 to the exon 21 of ENPP3 gene. H9C2 and AC-16 cells were transfected with shSRSF1. (C) RT-qPCR analysis of ENPP3 mRNA and lncRNA ENPP3 expression in cardiomyocytes. (D) The protein level of SRSF1 and ENPP3 was assessed by Western blotting. (E)&(F) The protein level of ENPP3 in the *in vivo* and* in vitro* models of CME was measured by Western blotting. (G)&(H) RIP assay validated the endogenous and exogenous binding of SRSF1 to ENPP3 pre-mRNA. (I) The wild type (WT) and mutant (MUT) ENPP3 splicing reporters containing SRSF1 binding sites were transfected into 293T cells. TRAP assay determined the interplay between SRSF1 and ENPP3 pre-mRNA. (J) H9C2 cells were transfected with shSRSF1 together with ENPP3 reporter-WT or ENPP3 reporter-MUT, and expression of ENPP3 mRNA/lncRNA and SRSF1 was assessed by RT-qPCR and Western blotting, respectively. H9C2 cells were transfected with SRSF1 overexpression plasmid together with ENPP3 reporter-WT or ENPP3 reporter-MUT. (K) The interaction between SRSF1 and ENPP3 mRNA was detected by RIP. (L) RT-qPCR and Western blotting analysis of ENPP3 mRNA/lncRNA and SRSF1 levels, respectively. For C, D, F-K, n=3. For E, n=6. Student's t test (for E, G) and one-way ANOVA (for C-F) were performed to analyze data. *p < 0.05, **p < 0.01, ***p < 0.001.

SRSF1 has been known as a crucial regulator of alternative splicing 15. Both StarBase and RBPDB databases predicted that SRSF1 could bind to ENPP3 (**Figure 2A**). As illustrated in **Figure 2B**, there are two transcripts of ENPP3 gene: one is ENPP3 mRNA (NM_005021.5) and the other is lncRNA ENPP3 (NR_133007.2) with Exon 21 skipping. To explore the regulation of SRSF1 in alternative splicing of ENPP3 gene, H9C2 and AC16 cells were transfected with shSRSF1#1, 2, 3, 4. The result indicated that shSRSF1#1 exhibited highest silencing efficiency (**Figure S1A, B**), and shSRSF1#1 was selected for the following experiments. The SRSF1 knockdown significantly decreased ENPP3 mRNA and protein expression but increased lncRNA ENPP3 levels in cardiomyocytes (**Figure 2C, D**). In addition, ENPP3 protein was highly expressed in CME rats and OGD-exposed cardiomyocytes (**Figure 2E, F**). The RIP assay confirmed that SRSF1 protein interacted with ENPP3 pre-mRNA both exogenously and endogenously (**Figure 2G, H**). Moreover, we constructed multiple ENPP3 plasmids containing WT/MUT binding sites to SRSF1. TRAP assay indicated that SRSF1 could bind to the WT-ENPP3, but not mutated plasmids of ENPP3 (**Figure 2I**). Silencing of SRSF1 reduced the ratio of ENPP3 mRNA to lncRNA ENPP3, which was counteracted by transfection with MUT-ENPP3 (**Figure 2J**). Additionally, the interplay between SRSF1 and ENPP3 mRNA disappeared in MUT-ENPP3 group (**Figure 2K**). Besides, mutation of ENPP3 partly reversed up-regulation of ENPP3 mRNA/lncRNA ENPP3 ratio in SRSF1 overexpressed cardiomyocytes (**Figure 2L**). Collectively, these results proved that SRSF1 directly bound to ENPP3 to facilitate its alternative splicing, leading to increased ratios of ENPP3 mRNA/lncRNA ENPP3.

### SRSF1 knockdown repressed CME-induced inflammation via affecting ENPP3 alternative splicing

**Figure 3:**
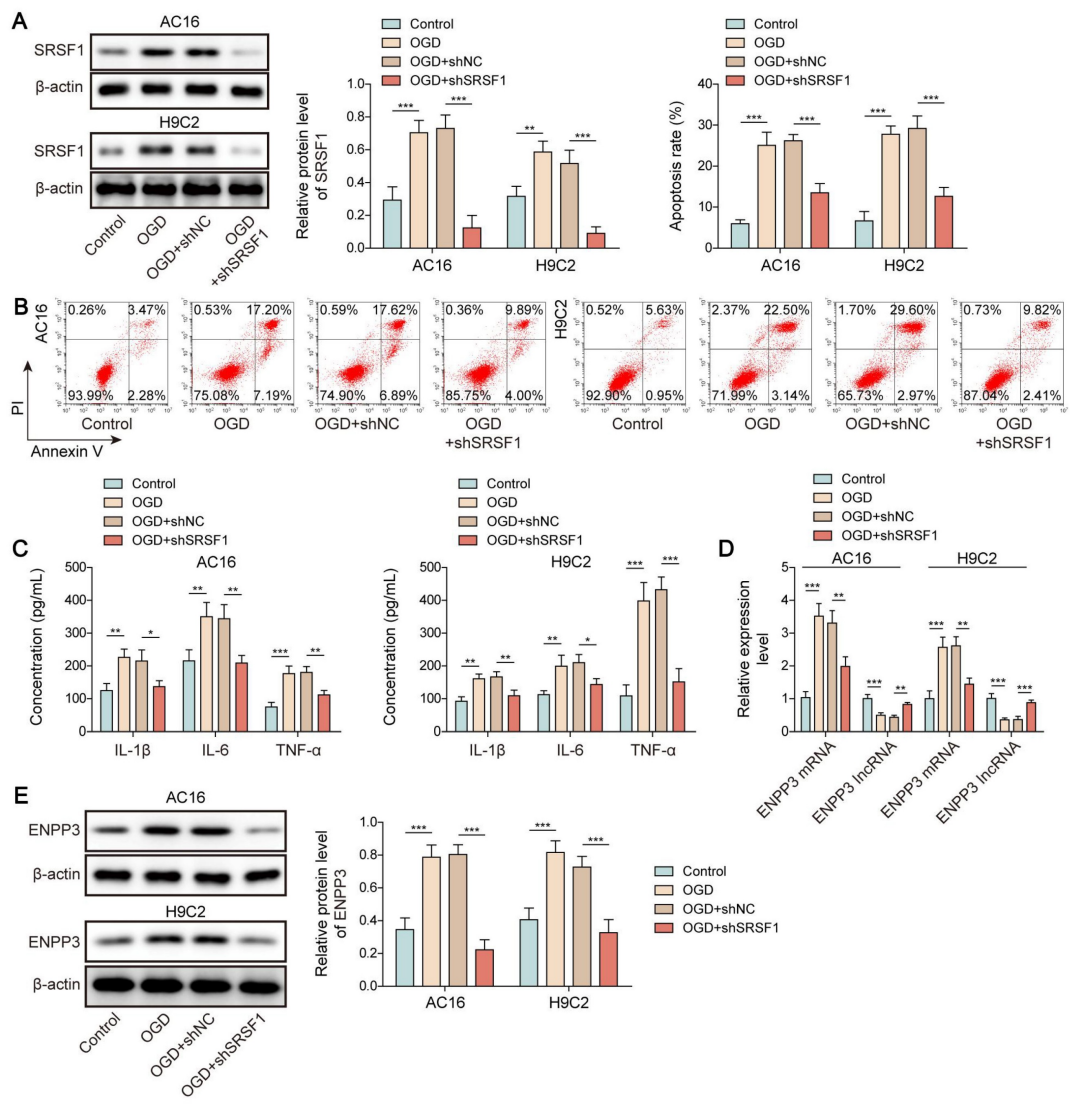
SRSF1 silencing suppressed cardiac inflammation in CME via modulation of ENPP3 splicing. H9C2 and AC-16 cells were transfected with shSRSF1, followed by stimulation with OGD. (A) Western blotting detected SRSF1 protein level. (B) Apoptosis of cardiomyocytes was detected by flow cytometry. (C) The production of TNF-α, IL-1β, and IL-6 was measured by ELISA. (D) RT-qPCR analysis of ENPP3 mRNA and lncRNA ENPP3 levels in each group. (E) The protein level of ENPP3 was determined by Western blotting. n=3 for A-E. One-way ANOVA was performed to analyze data. *p < 0.05, **p < 0.01, ***p < 0.001.

To determine the involvement of SRSF1-mediated ENPP3 alternative splicing in CME-induced inflammation, OGD-exposed cardiomyocytes were transfected with shSRSF1. The elevation of SRSF1 expression in OGD-challenged cardiomyocytes was reversed by transfection with shSRSF1 (**Figure 3A**). The reduction of SRSF1 expression blocked both OGD-induced apoptosis and the production of pro-inflammatory cytokines TNF-α, IL-1β and IL-6 in cardiomyocytes (**Figure 3B, C**). The up-regulation of ENPP3 mRNA and protein and down-regulation of lncRNA ENPP3 by OGD stimulation were blocked by SRSF1 depletion (**Figure 3D, E**). Taken together, our findings demonstrated that SRSF1 knockdown repressed inflammation during CME *in vitro* via regulation of ENPP3 alternative splicing.

### SRSF1 up-regulated ENPP3 to facilitate NF-κB p65-mediated transcription of pro-inflammatory cytokines

**Figure 4:**
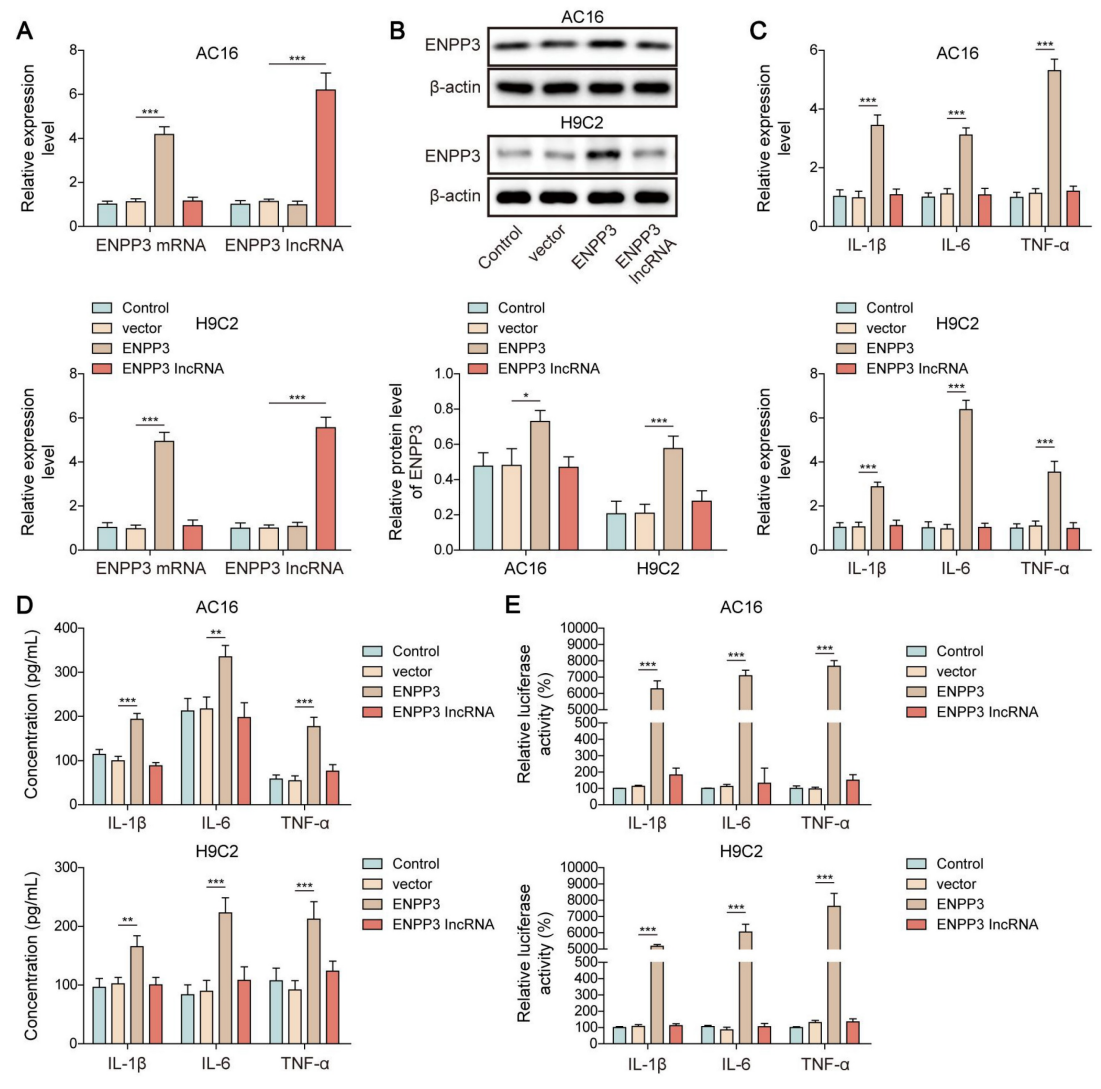

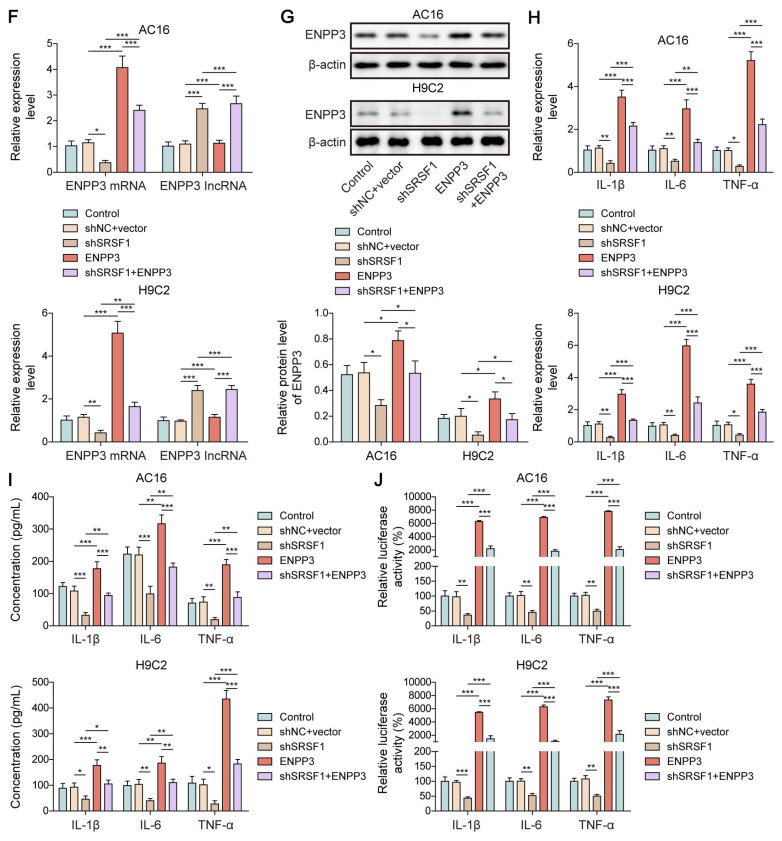
SRSF1 enhanced ENPP3 mRNA expression to trigger NF-κB p65-mediated transcription of pro-inflammatory cytokines. H9C2 and AC-16 cells were transfected with overexpression plasmid for ENPP3 or lncRNA ENPP3. (A) ENPP3 mRNA and lncRNA ENPP3 levels were evaluated by RT-qPCR. (B) Western blotting measured ENPP3 protein level in each group. (C) The mRNA levels of TNF-α, IL-1β, and IL-6 were assessed by RT-qPCR. (D) ELISA detected the release of TNF-α, IL-1β, and IL-6 from cardiomyocytes. (E) The binding of NF-κB p65 to the promoters of TNF-α, IL-1β, and IL-6 was determined by dual-luciferase reporter assay. H9C2 and AC-16 cells were transfected with shSRSF1, overexpression plasmid for ENPP3, or a combination of them. (F) ENPP3 mRNA and lncRNA ENPP3 levels were measured by RT-qPCR. (G) Western blotting analysis of ENPP3 protein level in cardiomyocytes. (H)&(I) The levels of TNF-α, IL-1β, and IL-6 in cardiomyocytes were evaluated by RT-qPCR and ELISA. (J) Dual-luciferase reporter assay analyzed the interaction between NF-κB p65 and the promoters of TNF-α, IL-1β, and IL-6. n=3 for A-J. One-way ANOVA was performed to analyze data. *p < 0.05, **p < 0.01, ***p < 0.001.

NF-κB p65 functions as a well-established transcriptional factor which activates the transcription and expression of pro-inflammatory cytokines 16. Thus, we further evaluated the role of ENPP3 alternative splicing in NF-κB p65-mediated myocardial inflammation. For this purpose, we overexpressed ENPP3 mRNA and lncRNA ENPP3 in H9C2 and AC16 cells, respectively (**Figure 4A, B**). ENPP3 mRNA overexpression in cardiomyocytes led to significant increases in TNF-α, IL-1β and IL-6 mRNA expression and production but lncRNA ENPP3 overexpression did not produce this effect (**Figure 4C, D**). ENPP3 mRNA overexpression strengthened NF-κB p65 transcription of TNF-α, IL-1β and IL-6 but lncRNA ENPP3 overexpression did not produce this effect (**Figure 4E**). The study used rescue experiments to validate the regulation of SRSF1/ENPP3 axis in NF-κB p65-mediated inflammation by transfection with shSRSF1, ENPP3 overexpression plasmid, or a combination of them. ENPP3 overexpression restored SRSF1 silencing-mediated decreased expression of ENPP3, whereas the increased expression of lncRNA ENPP3 was not changed by ENPP3 overexpression (**Figure 4F, G**). ENPP3 overexpression eliminated the suppressive impact of SRSF1 deficiency on TNF-α, IL-1β and IL-6 transcription and expression and release (**Figure 4H-J**). These observations revealed that NF-κB p65-mediated transcription of pro-inflammatory cytokines was repressed by inactivation of SRSF1/ENPP3 axis.

### O-GlcNAcylation of BRD4 restrained NF-κB p65-mediated transcription of pro-inflammatory cytokines

**Figure 5:**
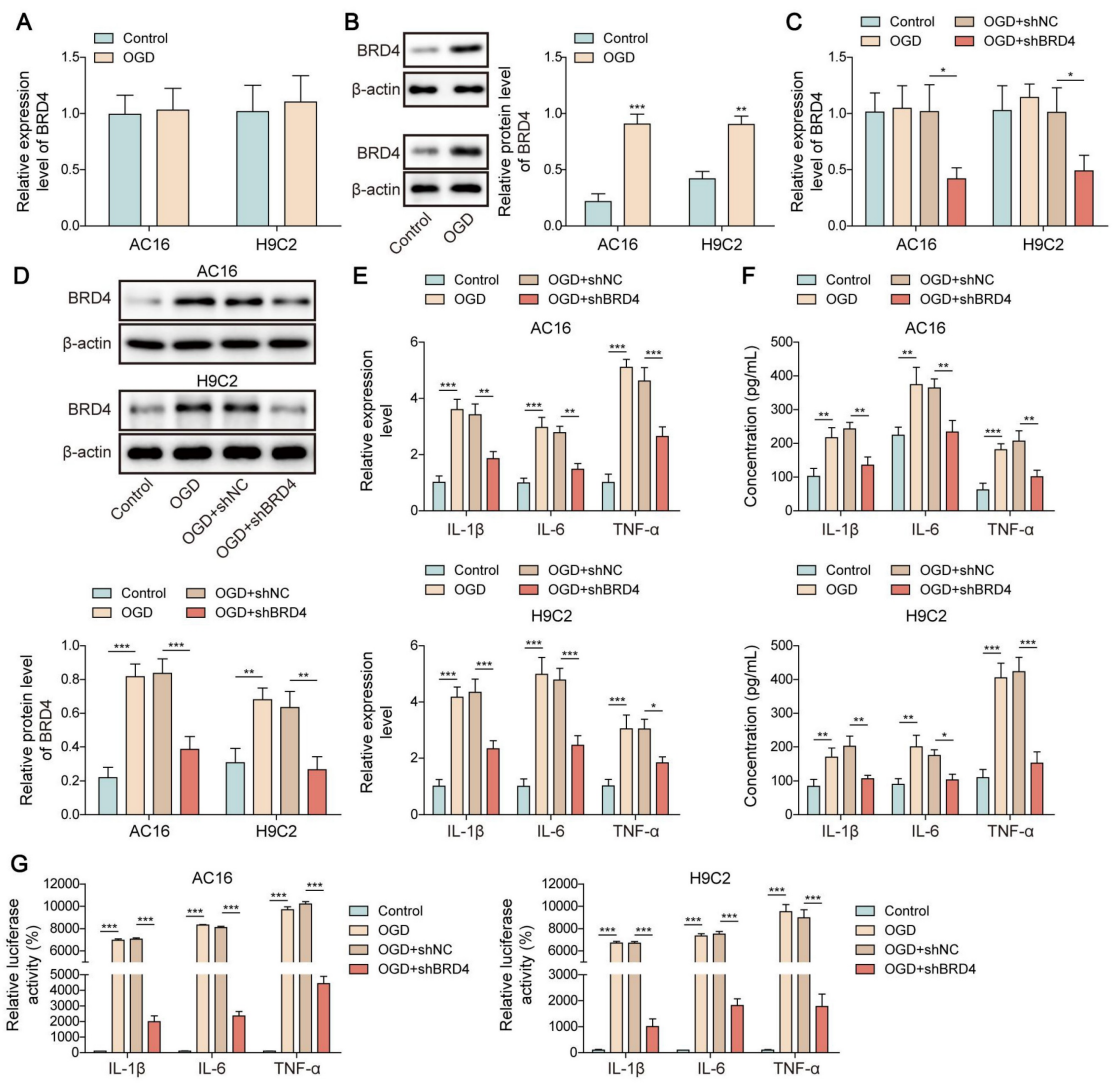

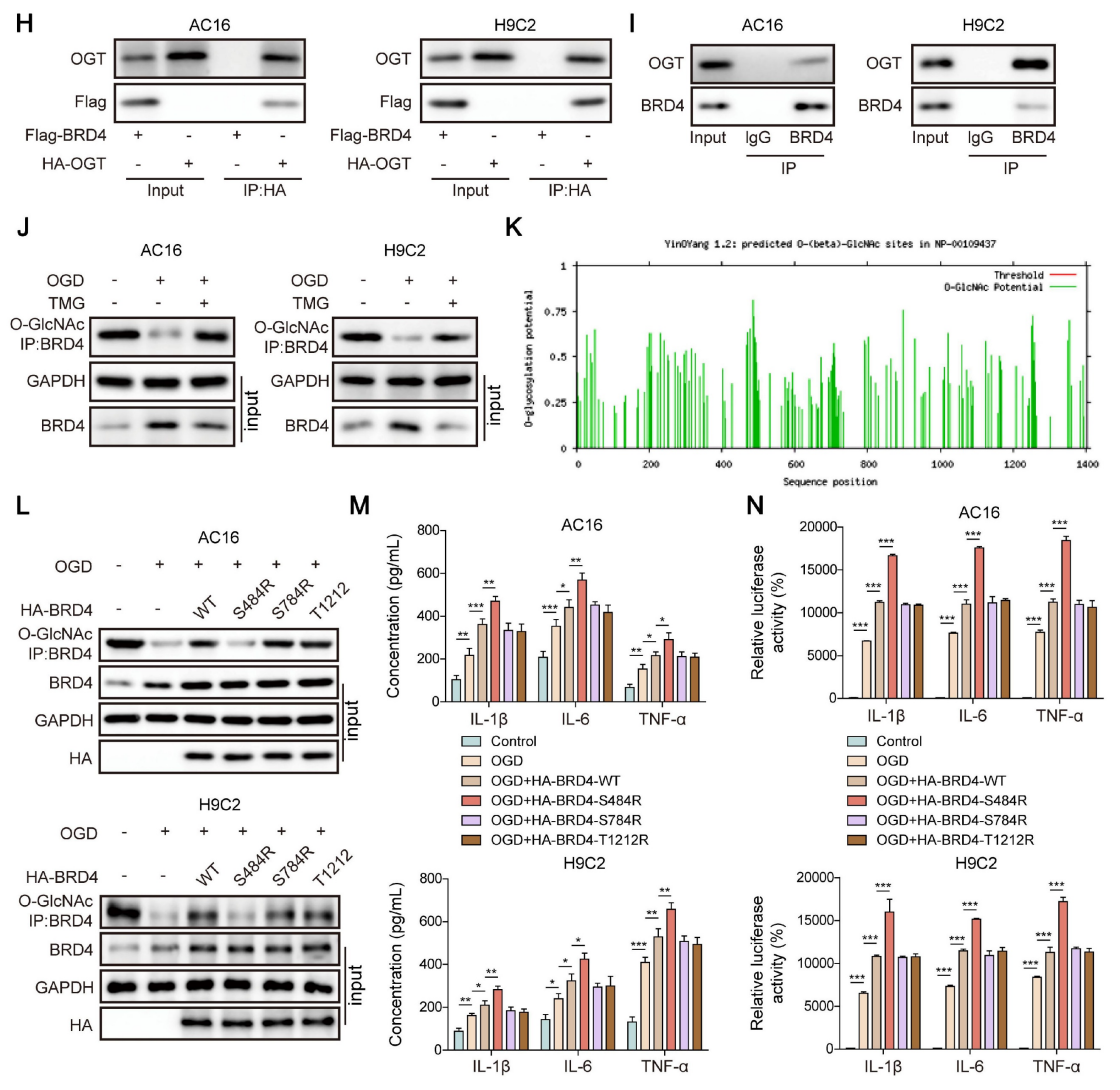
O-GlcNAcylation of BRD4 inhibited NF-κB p65-mediated transcription of pro-inflammatory cytokines. (A)&(B) The expression of BRD4 in OGD-exposed cardiomyocytes was detected by RT-qPCR and Western blotting. H9C2 and AC-16 cells were transfected with shBRD4, and then subjected to OGD. (C)&(D) RT-qPCR and Western blotting analysis of BRD4 mRNA and protein levels. (E)&(F) The mRNA levels and concentrations of TNF-α, IL-1β, and IL-6 were determined by RT-qPCR and ELISA. (G) The binding of NF-κB p65 to TNF-α, IL-1β, and IL-6 promoters was confirmed by dual-luciferase reporter assay. (H)&(I) Co-IP assay verified the exogenous and endogenous interplay between OGT and BRD4 proteins. (J) O-GlcNAcylation of BRD4 protein in OGD-stimulated cardiomyocytes was evaluated. (K) YinOYang database predicated the potential O-GlcNAc sites on BRD4. OGD-challenged H9C2 and AC-16 cells were transfected with BRD4 WT plasmid or BRD4 plasmids with mutant O-GlcNAc sites (BRD4-S484R, BRD4-S784R, and BRD4-T1212R). (L) O-GlcNAcylation of BRD4 protein in H9C2 and AC-16 cells was detected. (M) Concentrations of TNF-α, IL-1β, and IL-6 were detected by ELISA. (N) The interaction between NF-κB p65 and TNF-α, IL-1β, and IL-6 promoters was validated by dual-luciferase reporter assay. n=3 for A-N. Student's t test (for A, B) and one-way ANOVA (for C-G, M, N) were performed to analyze data. *p < 0.05, **p < 0.01, ***p < 0.001.

To further elucidate the downstream mechanism through which SRSF1/ENPP3 axis modulated NF-κB p65-mediated transcription of pro-inflammatory cytokines, we focused on BRD4. BRD4 has been identified to be a crucial activator of NF-κB p65-mediated inflammation 17. Interestingly, the protein level of BRD4 was remarkably increased by OGD challenge in cardiomyocytes, whereas the mRNA level of BRD4 was not changed (**Figure 5A, B**). The silencing efficiency of shBRD4#1, 2, 3, 4 was detected by RT-qPCR. The highest silencing efficiency was observed in shBRD4#1 as shown in **Figure S1C**. The shBRD4 plasmid was transfected into OGD-exposed cardiomyocytes to knockdown BRD4 expression (**Figure 5C, D**). The mRNA expression and pro-inflammatory cytokine production in OGD-exposed cardiomyocytes was suppressed by BRD4 ablation (**Figure 5E, F**). The binding of NF-κB p65 to the promoters of TNF-α, IL-1β, and IL-6 was enhanced by OGD stimulation, which was reduced by shBRD4 (**Figure 5G**). The interaction between O-GlcNAc transferase (OGT) and BRD4 proteins was confirmed by Co-IP assay (**Figure 5H, I**). In addition, the O-GlcNAc level of BRD4 was evidently reduced by OGD exposure, which could be recovered by treatment with Thiamet-G (TMG, O-GlcNAcase inhibitor) (**Figure 5J**). There were several potential O-GlcNAc sites (S484, S784, T1212) in BRD4 protein (**Figure 5K**). To identify the exact O-GlcNAc sites of BRD4, cardiomyocytes were transfected with multiple BRD4 plasmids with WT or mutant O-GlcNAc sites (BRD4-S484R, BRD4-S784R, and BRD4-T1212R). We found that transfection with BRD4-WT enhanced the O-GlcNAc level of BRD4 in OGD-stimulated cardiomyocytes, which was abolished by only BRD4-S484R (**Figure 5L**), suggesting that BRD4 was O-GlcNAcylated at the S484 site. Furthermore, mutation at the S484 site further enhanced the promotive effects of BRD4-WT on the release and transcription of TNF-α, IL-1β, and IL-6 in OGD-exposed cardiomyocytes (**Figure 5M, N**). Therefore, O-GlcNAcylation of BRD4 at the S484 site exerted inhibitory effect on transcription and production of pro-inflammatory cytokines.

### ENPP3 suppressed O-GlcNAcylation of BRD4 to trigger inflammation

**Figure 6:**
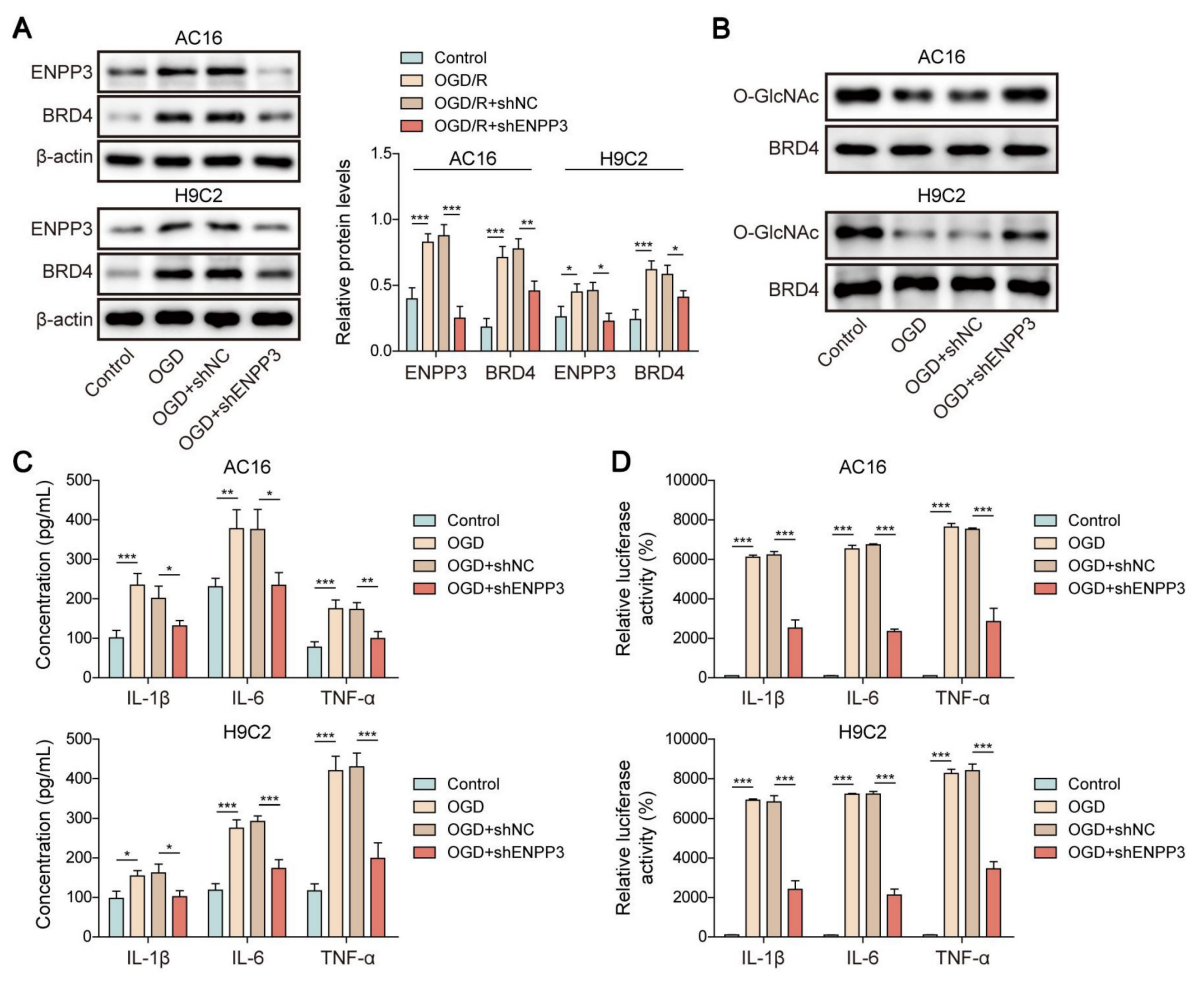
ENPP3 contributed to inflammation by inhibiting O-GlcNAcylation of BRD4. H9C2 and AC-16 cells were transfected with shENPP3, followed by exposure to OGD. (A) ENPP3 and BRD4 protein levels were measured by Western blotting. (B) The O-GlcNAc level of BRD4 protein was assessed. (C) The production of TNF-α, IL-1β, and IL-6 was determined by ELISA. (D) Dual-luciferase reporter assay evaluated the binding of NF-κB p65 to TNF-α, IL-1β, and IL-6 promoters. n=3 for A-D. One-way ANOVA was performed to analyze data. *p < 0.05, **p < 0.01, ***p < 0.001.

We next examined the influence of ENPP3 on O-GlcNAcylation of BRD4 in the *in vitro* model of CME. RT-qPCR analyzed the silencing efficiency of shENPP3#1, 2, 3, 4. shENPP3#2 with highest silencing efficiency was adopted (**Figure S1D**). Then, shENPP3 was transfected into H9C2 and AC16 cells upon OGD stimulation. The enhanced protein level of BRD4 was reversed by shENPP3 transfection in OGD-stimulated cardiomyocytes (**Figure 6A**). Additionally, ENPP3 knockdown effectively restored the reduced O-GlcNAc level of BRD4 in OGD-exposed cardiomyocytes (**Figure 6B**). Besides, the increased expression and transcription of TNF-α, IL-1β, IL-6 after OGD exposure were abrogated in ENPP3-depleted cardiomyocytes (**Figure 6C, D**). These results proved that ENPP3 contributed to CME-induced inflammation via repressing O-GlcNAcylation of BRD4.

### SRSF1 facilitated inflammation in CME through ENPP3-mediated inhibition of BRD4 O-GlcNAcylation

**Figure 7:**
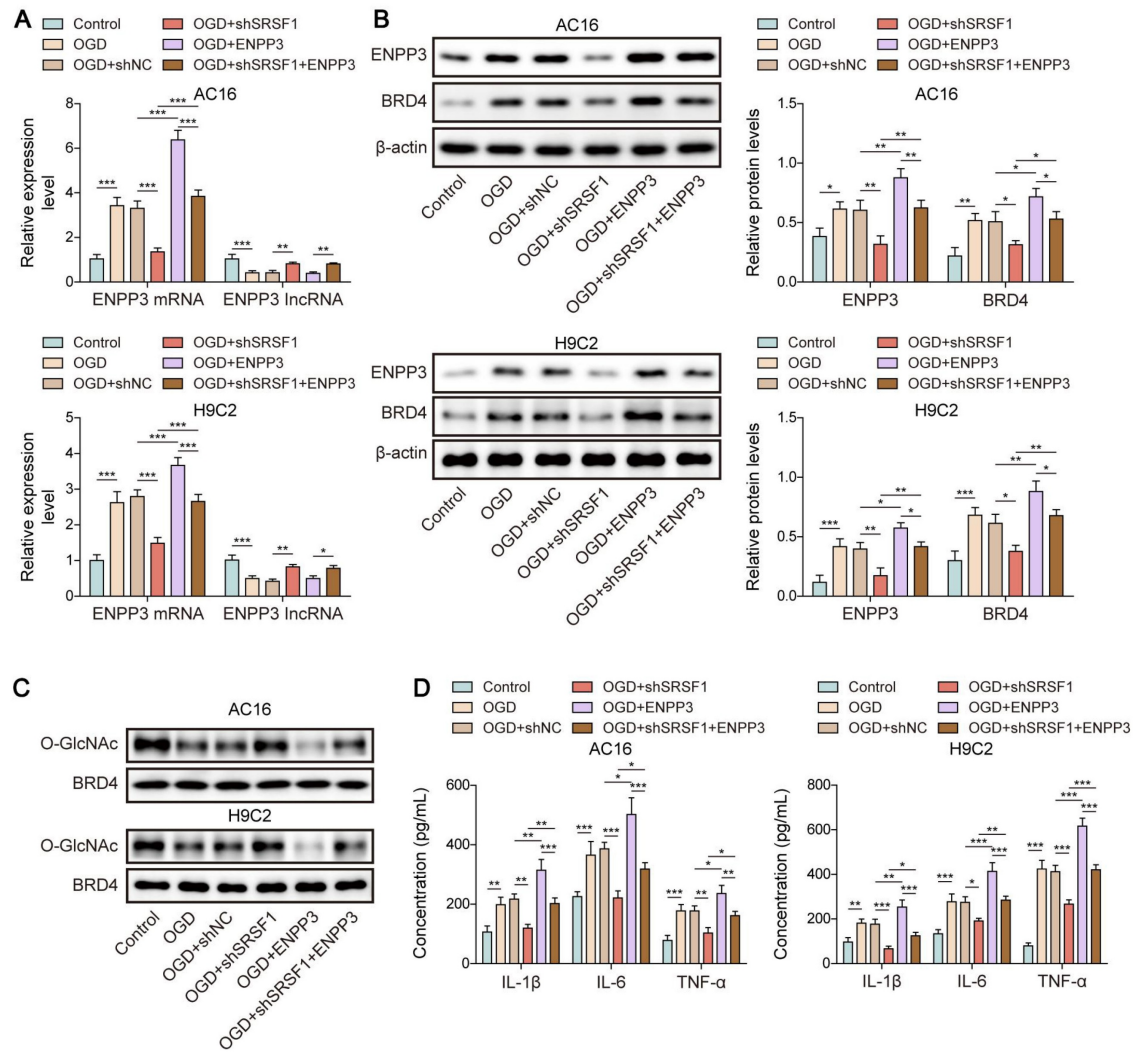
SRSF1/ENPP3 axis suppressed BRD4 O-GlcNAcylation to promote inflammation in CME. The OGD-stimulated cardiomyocytes were transfected with shSRSF1, ENPP3 overexpression plasmid, or a combination of them. (A) ENPP3 mRNA and lncRNA ENPP3 expression levels were detected by RT-qPCR. (B) The protein abundance of ENPP3 and BRD4 was assessed by Western blotting. (C) The O-GlcNAc level of BRD4 was determined. (D) ELISA was carried out to measure TNF-α, IL-1β, and IL-6 concentrations. n=3 for A-D. One-way ANOVA was performed to analyze data. *p < 0.05, **p < 0.01, ***p < 0.001.

To further verify the modulation of SRSF1/ENPP3 axis in O-GlcNAcylation of BRD4, OGD-exposed cardiomyocytes were transfected with shSRSF1, ENPP3 overexpression plasmid, or a combination of them. ENPP3 overexpression could abolish SRSF1 deficiency-mediated down-regulation of ENPP3 and BRD4; however, shSRSF1-mediated up-regulation of lncRNA ENPP3 was not affected by ENPP3 overexpression (**Figure 7A, B**). Moreover, SRSF1 silencing restored the reduced O-GlcNAc level of BRD4 upon OGD stimulation, which was abolished by ENPP3 overexpression (**Figure 7C**). Furthermore, the release of TNF-α, IL-1β, IL-6 from OGD-stimulated cardiomyocytes was suppressed by SRSF1 depletion, and the inhibitory effect of SRSF1 ablation was neutralized by enforced expression of ENPP3 (**Figure 7D**). To sum up, activation of SRSF1/ENPP3 axis repressed O-GlcNAcylation of BRD4 to trigger inflammation in CME.

### Myocardium-specific SRSF1 knockout attenuated inflammation in CME via modulation of ENPP3/BRD4/NF-κB axis

**Figure 8:**
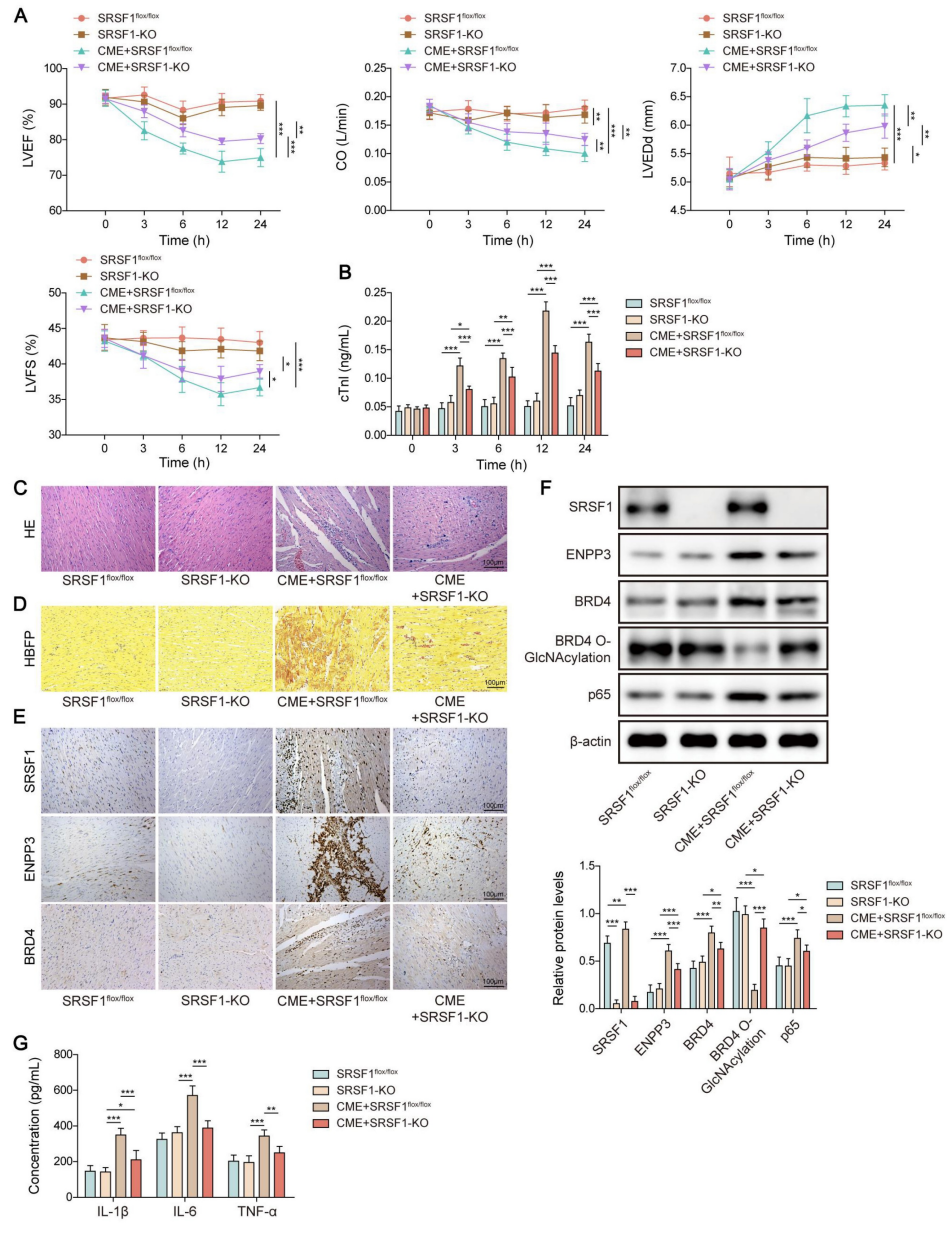
Myocardium-specific SRSF1 knockout alleviated CME-induced inflammation via inactivation of the ENPP3/BRD4/NF-κB pathway. SRSF1^flox/flox^ and SRSF1-KO rats were injected with microspheres into the left ventricle to induce CME. (A) LVEF, LVFS, LVEDd, and CO were detected to evaluate cardiac function. (B) The serum cTnl level in different groups was measured by ELISA. (C) Pathological alterations in myocardial tissues were observed by HE staining (scale bar = 100 μm). (D) Myocardial infarct size was measured by HBFP staining (scale bar = 100 μm). (E) SRSF1, ENPP3, and BRD4 expression in myocardial tissues was evaluated by immunohistochemical staining (scale bar = 100 μm). (F) The protein abundance of SRSF1, ENPP3, BRD4, p65, and O-GlcNAcylation of BRD4 was detected by Western blotting or Co-IP, respectively. (G) ELISA was carried out to measure TNF-α, IL-1β, and IL-6 concentrations. n=6 for A-G. ANOVA for repeated measurement (for A, B), and one-way ANOVA (for F, G) was performed to analyze data. *p < 0.05, **p < 0.01, ***p < 0.001.

Finally, we validated the *in vivo* biological function of SRSF1 in inflammation during CME. Myocardial dysfunction of CME rats was attenuated by myocardium-specific SRSF1 knockout as evidenced by increasing LVEF, LVFS, and CO levels, and decreasing LVEDd level (**Figure 8A**). In addition, SRSF1 deficiency ameliorated myocardial damage via reducing serum cTnI level (**Figure 8B**). Accordingly, the pathological changes in the heart tissues of CME rats were attenuated after SRSF1 knockout (**Figure 8C**). HBFP staining indicated that SRSF1 knockout did not affect the percentage of HBFP-positive cells. However, SRSF1 knockout remarkably reduced the elevation of HBFP-positive cell percentage in CME rats (**Figure 8D**). Immunohistochemical staining indicated that SRSF1 knockout reversed the increased expression of SRSF1, ENPP3 and BRD4 in the myocardial tissues of CME rats (**Figure 8E**). Consistently, the enhanced protein levels of ENPP3, BRD4 and NF-κB p65, and the decreased O-GlcNAc level of BRD4 were partly reversed by myocardium-specific SRSF1 knockout (**Figure 8F**). Besides, SRSF1 knockout counteracted the elevated serum levels of TNF-α, IL-1β, and IL-6 of CME rats (**Figure 8G**). Collectively, myocardium-specific SRSF1 knockout restrained inflammation during CME in rats through regulation of ENPP3/BRD4/NF-κB pathway.

## Discussion

As a major complication of acute coronary syndrome, CME leads to serious myocardial dysfunction via inducing inflammation and apoptosis 18. Inflammation has been considered as a crucial driver of myocardial dysfunction due to CME 19. Splicing factor SRSF1 has been recognized to affect autoimmunity and tissue inflammation through regulation of effector T cells 20. This study for the first time investigated the biological function and underlying mechanisms of SRSF1 in CME-induced myocardial inflammation. Our findings revealed that SRSF1 facilited alternative splicing of ENPP3 pre-mRNA to enhance ENPP3 mRNA expression, which subsequently inhibited O-GlcNAcylation of BRD4 to facilitate NF-κB p65-mediated myocardial inflammation during CME.

Local myocardial inflammatory response triggered by CME has been verified to be a key contributor to cardiac dysfunction. Research has confirmed that inflammatory cells infiltrate the myocardium while pro-inflammatory factors accumulate during CME-induced myocardial microinfarction 21. The NF-κB pathway becomes overactive to produce excessive amounts of pro-inflammatory cytokines (TNF-α, IL-1β, IL-6), while inactivation of NF-κB pathway can relieve inflammation and improve cardiac function 22. Besides cardiac inflammation, CME may cause energy metabolism disturbances. The citrate cycle pathway showed significant energy metabolic abnormalities in CME mice according to previous research 23. The KEGG pathway analysis showed that the metabolism upstream modulator PPAR signaling pathway was enriched in CME mice 23, suggesting energy metabolic dysfunction in CME. In this study, SRSF1 was abnormally up-regulated in CME models, and SRSF1 knockdown effectively repressed CME-induced myocardial inflammation and apoptosis.

Alternative pre-mRNA splicing has been identified to take part in multiple physio-pathological processes, including inflammation 24. So far, the influence of alternative pre-mRNA splicing on CME has not been clarified. The serine-arginine (SR) proteins of splicing factor family initiate the spliceosome assembly and activation process and control RNA metabolism through alternative splicing 25. The SRSF1 protein functions as a member of this family to either activate or suppress mRNA splicing processes. SRSF1 regulates cyclin D1 alternative splicing during mechanical stress according to Feng *et al.* which results in uncontrolled cell proliferation 26. Research shows that SRSF1 promotes exon 10 inclusion during tau alternative splicing in Alzheimer's disease 27. In this work, SRSF1 level was found to be elevated after CME induction. Interestingly, SRSF1 could directly bind to the pre-mRNA of ENPP3, it is therefore possible that SRSF1 might regulate the pre-mRNA splicing of ENPP3 pre-mRNA. ENPP3 gene can produce two functionally diverse transcripts, ENPP3 mRNA (NM_005021.5, containing 27 exons) and lncRNA ENPP3 (NR_133007.2, skipping of alternative exon 21). ENPP3, also known as CD203c, is an important basophil activation marker that plays critical roles in chronic allergic inflammation 28. Previous study has shown that ENPP3 was a target gene of miR-34a-5p, which mediated microglia inflammation caused by diabetic neuropathic pain 29. In our study, we discovered that SRSF1 promoted ENPP3 pre-mRNA alternative splicing shift from lncRNA ENPP3 to ENPP3 mRNA in cardiomyocytes. In line with previous studies, SRSF1 promoted NF-κB p65-mediated pro-inflammatory cytokine transcription via enhancing ENPP3 mRNA/lncRNA ENPP3 ratio, resulting in inflammation during CME progression.

To further explore the upstream modulatory mechanism of NF-κB p65-mediated inflammation in CME, BRD4 was focused on. BRD4 has been demonstrated to be a modulator of NF-κB in various diseases 30, 31. Notably, BRD4 was up-regulated in cardiac hypertrophy model, and BRD4 inhibition could effectively attenuate cardiac hypertrophy 32. So far, the biological function of BRD4 in CME has not been determined. Our study found an elevation in protein level of BRD4 during CME, whereas the mRNA level of BRD4 was not altered. Thus, BRD4 might be regulated via post-translational mechanism in CME. Of note, there were several potential O-GlcNAc sites on BRD4, suggesting that BRD4 might be regulated by O-GlcNAcylation. The post-translational modification known as O-GlcNAcylation involves the attachment of N-acetylglucosamine (GlcNAc) to serine or threonine residues in proteins. The reversible regulation of O-GlcNAcylation occurs through the actions of two primary enzymes: O-GlcNAc transferase (OGT) and O-GlcNAcase (OGA) 33. As reported by Dong *et al.*, OGT-mediated O-GlcNAcylation of NF-κB p65 protected against inflammatory activation of astrocytes 14. A recent study suggested that enhancement of O-GlcNAcylation of COX10 protected against myocardial ischemia-reperfusion injury via attenuating mitochondrial damage 34. Consistent with the previous study, we found a reduced O-GlcNAc level during CME induction, and BRD4 was GlcNAcylated at the S484 site in cardiomyocytes. To further examine the influence of O-GlcNAcylation of BRD4 on CME-induced inflammation, we mutated a series of O-GlcNAc sites. Mutation at the S484 site further promoted NF-κB p65-mediated release of TNF-α, IL-1β, IL-6. As reported, ENPP3 could hydrolyze UDP-GlcNAc, the only donor substrate for O-GlcNAc, which suggested the inhibitory role of ENPP3 in GlcNAcylation 10. Our observations demonstrated that ENPP3 restrained O-GlcNAcylation of BRD4 to intensify CME-induced inflammation. Cumulatively, our observations indicated that O-GlcNAcylation of BRD4 was inhibited by ENPP3 during CME, thus enhancing BRD4 protein level to trigger NF-κB-mediated inflammation.

Finally, we performed *in vitro* and *in vivo* tests to validate the regulation of SRSF1/ENPP3 axis in O-GlcNAcylation of BRD4 protein. Our data proved that knockdown of SRSF1 enhanced the O-GlcNAc level of BRD4 via down-regulation of ENPP3, thereby inhibiting inflammation to improve cardiac function during CME. However, further investigation is needed to explore the detailed mechanism through which ENPP3 restrained O-GlcNAcylation of BRD4.

There are several limitations in this study. First, myocardial cell lines instead of primary cardiomyocytes were used to establish the CME model* in vitro*. We need to further validate the* in vitro* results in primary cardiomyocytes. Second, cellular energy metabolism disturbance has been validated after CME, future studies are warranted to clarify the role of SRSF1 in energy metabolism in CME model. Third, although the mouse model functions as a standard tool to study human disease development, further investigations of our findings in human cardiac tissues with CME should be taken into account.

In conclusion, abnormal high expression of SRSF1 facilitated CME-induced cardiac inflammation via promoting NF-κB-mediated transcription and production of inflammatory cytokines. Mechanistically, SRSF1 modulated alternative splicing of ENPP3 pre-mRNA to enhance ENPP3 mRNA/lncRNA ENPP3 ratio, which raised BRD4 protein level via inhibiting its O-GlcNAcylation at the S484 site. Our research provides essential knowledge about SRSF1 regulatory functions in CME-induced cardiac inflammation which could result in discovering therapeutic targets for CME-induced myocardial dysfunction prevention and treatment.

## Materials and Methods

### Animal studies

Male SRSF1^flox/flox^ rats and α-MHC-Cre transgenic rats on Sprague-Dawley background (eight weeks old, 250-300 g) were generated by Cyagen (Suzhou, China) according to a previous study 35. To obtain myocardium-specific SRSF1 knockout rats (named SRSF1-KO), the α-MHC-Cre rats were crossed with the SRSF1^flox/flox^ rats. SRSF1^flox/flox^ rats were used as control rats. There were four experimental groups (n=6 per group): SRSF1^flox/flox^, SRSF1-KO, CME+SRSF1^flox/flox^, CME+SRSF1-KO. To establish CME model, the SRSF1^flox/flox^ and SRSF1-KO rats received pentobarbital anesthesia through intraperitoneal administration at a dose of 60 mg/kg. After thoracotomy, the ascending aorta was exposed, followed by occlusion by a vascular clamp for 10 s. The left ventricle received 3000 microspheres (42 μm in diameter, Biosphere Medical, Rockland, USA) through the apex in 0.1 mL of normal saline. The sham rats received 0.1 mL of phosphate buffer saline (PBS) through injection. Thereafter, the chest was closed, followed by 20,000 U/kg penicillin injection to avoid infection. The serum samples and heart tissues were obtained from the euthanized rats at 24 h after CME. The ethics committee at Guilin Medical University approved all animal procedures under the approval number GLMC202203014.

### Evaluation of cardiac function

The cardiac function of rats was assessed at 3, 6, 12, and 24 h following the induction of CME. The Hewlett Packard Sonos 7500 (Philips, Andover, Mass, USA) was utilized to measure the left ventricular ejection fraction (LVEF), left ventricular end-diastolic diameter (LVEDd), left ventricular fractional shortening (LVFS), and cardiac output (CO) throughout three cardiac cycles.

### Haematoxylin and eosin (HE) staining

The heart tissues underwent preservation in 4% paraformaldehyde before being embedded in paraffin for sectioning into 4-μm slices. Standard HE staining was performed on the cardiac sections using the commercial HE Staining Kit (Sangon, Shanghai, China). The pathogenic changes in the cardiac sections were analyzed microscopically (Olympus, Tokyo, Japan).

### Hematoxylin-basic fuchsin-picric acid (HBFP) staining

The heart sections were dewaxed and rehydrated. After nuclear staining with hematoxylin, the sections were stained with 0.1% basic fuchsin, differentiated with 0.1% picric acid for 15 s, and then washed with acetone. The stained specimens were examined under a microscope.

### Immunohistochemical staining

The heart sections received dewaxing treatment followed by rehydration and antigen retrieval through citrate buffer application. The sections received overnight incubation with primary antibodies SRSF1 (12929-2-AP, 1:50, Proteintech, Wuhan, China), ENPP3 (A05615, 1:100, Boster, CA, USA), or BRD4 (M00123, 1:50, Boster) at 4 °C. The following day the sections received a 1:50 dilution of HRP Goat Anti-Rabbit IgG (H+L) (AS014, ABclonal). The positive staining became visible after a 5-minute exposure to 3,3′-diaminobenzidine (DAB). The sections underwent hematoxylin counterstaining before being analyzed through a light microscope.

### Cell culture and treatment

The H9C2 rat cardiomyocytes (CRL-1446, ATCC, USA) and human cardiomyocytes AC16 (CL-0790, Procell, Wuhan, China) were cultured in DMEM (Thermo Fisher, MA, USA) with 10% fetal bovine serum (FBS, Thermo Fisher) at 37 °C in a 5% CO_2_ atmosphere. The CME *in vitro* model was created by exposing H9C2 and AC16 cells to oxygen-glucose deprivation (OGD) through serum-free DMEM culture at 37 °C with 5% CO_2_ and 95% N_2_ for 2, 4, 8 and 12 h. The control cells were maintained under standard culture conditions.

### Cell transfection

Short hairpin RNA targeting SRSF1 (shSRSF1 #1, 2, 3, 4), shBRD4 #1, 2, 3, 4, shENPP3 #1, 2, 3, 4, and negative control shRNA (shNC), vector, overexpression plasmids for ENPP3, ENPP3 lncRNA, Flag-SRSF1, Flag-vector, ENPP3 plasmid containing wild type/mutant binding sites to SRSF1 (ENPP3 reporter-WT/ENPP3 reporter-MUT), BRD4 plasmid with mutant GlcNAcylation sites (BRD4-S484R, BRD4-S784R, and BRD4-T1212R) were provided by GenePharm (Shanghai, China). The ShRNA sequences are shown in **Table S1**. The cells were transfected with the constructed plasmids or shRNAs using Lipofectamine 2000 (Thermo Fisher) for 48 h after being inoculated into 6-well plates (4×10^5^ cells per well).

### Enzyme-linked immunosorbent assay (ELISA)

The concentrations of cardiac troponin I (cTnI), tumor necrosis factor-α (TNF-α), Interleukin-1β (IL-1β), and Interleukin-6 (IL-6) were quantified utilizing commercial ELISA kits for cTnI (Cat. No. SEA478Ra), TNF-α (Cat. No. SEA133Hu & HEA133Ra), IL-1β (Cat. No. SEA563Hu & SEA563Rb), and IL-6 (Cat. No. SEA079Hu & SEA079Ra) acquired from USCN (Wuhan, China), in accordance with the manufacturer's guidelines.

### Real-time quantitative PCR (RT-qPCR)

The TRIzol reagent (Thermo Fisher) was used to extract total RNA from H9C2 and AC16 cells. Total RNA was converted to cDNA by reverse transcription using the RevertAid First Strand cDNA Synthesis Kit (Cat. No. K1622, Thermo Fisher). RT-qPCR was performed using the SYBR Green Master Mix (Cat. No. A46110 Thermo Fisher) with specific gene primers. The relative gene expression levels normalized to GAPDH were determined using the 2^-ΔΔCT^ technique. The primer sequences are included in **Table S2**.

### Western blotting

The extraction of total protein was performed using refrigerated RIPA buffer from Beyotime (Haimen, China). The BCA Protein Assay Kit (Sangon) was used to evaluate protein content after modification. The protein samples were separated by SDS-PAGE and then transferred to polyvinylidene fluoride membranes before blocking with 5% skimmed milk. The membranes were treated overnight at 4 °C with primary antibodies targeting SRSF1 (12929-2-AP, 1:1000, Proteintech), ENPP3 (A05615, 1:1000, Boster), BRD4 (M00123, 1:500, Boster), p65 (ab32536, 1:1000, Abcam), β-tubulin (ab6046, 1:1000, Abcam), and β-actin (AC038, 1:10000, ABclonal, Wuhan, China). The membranes were then treated with HRP Goat Anti-Rabbit IgG (AS014, 1:2000, ABclonal). The protein bands were observed using the ECL luminescence reagent (Sangon) and quantified with ImageJ software (NIH, MA, USA).

### Flow cytometry

The cardiomyocytes underwent preservation in ice-cold 70% ethanol throughout an overnight period. The cardiomyocytes received 10 μL of annexin V-FITC solution for staining purposes. The cells underwent a 15-minute incubation period in darkness before receiving 5 μL of propidium iodide (PI) dye with RNase (Beyotime Biotechnology, Shanghai, China). The flow cytometer (Becton-Dickinson, New Jersey, USA) was used to measure apoptotic cell numbers.

### RNA immunoprecipitation (RIP) assay

The SRSF1 and ENPP3 pre-mRNA interaction was validated through RNA Immunoprecipitation (RIP) with the EZ-Magna RIP RNA-Binding Protein Immunoprecipitation Kit (Millipore; MA, USA). The cells undergoing different transfection procedures received protease and RNase inhibitor treatment during lysis. The cell lysates underwent overnight incubation at 4 °C with magnetic beads that were either anti-Flag (ab205606, Abcam) or anti-SRSF1 (12929-2-AP, Proteintech) or anti-IgG (ab172730, Abcam). The extracted RNAs underwent purification before RT-PCR analysis.

### Tagged RNA affinity purification (TRAP)

TRAP assay was conducted to verify the binding between ENPP3 pre-mRNA and SRSF1 protein. MS2-binding sequences (MS2bs) were inserted into ENPP3 pre-mRNA overexpressing vectors containing wild type/mutant binding sites to SRSF1. 293T cells were co-transfected with 6 μg of MS2-ENPP3 pre-mRNA plasmid or MS2-vector plasmid together with 2 μg of GST-MS2 plasmid. After transfection for 48 h, the cell lysates were pulled down with the glutathione (GSH) agarose beads overnight at 4 °C, and then washed with NT2 buffer. SRSF1 protein level in ENPP3 pre-mRNA-binding proteins was detected by Western blotting.

### Dual luciferase reporter assay

The wildtype (WT) and mutant (MUT) sequences of the TNF-α, IL-1β, and IL-6 promoter regions, which include binding sites for NF-κB p65, were subcloned into the pGL3 vector (Promega, WI, USA) to create luciferase reporter vectors. OGD-stimulated H9C2 and AC16 cells were co-transfected with the engineered luciferase reporter vectors alongside shNC or shENPP3. Luciferase activity was assessed 48 h post-transfection utilizing the Dual-Luciferase Reporter Assay kit (Promega).

### Co-immunoprecipitation (Co-IP)

Co-IP assay was performed to confirm the exogenous and endogenous interaction between OGT and BRD4 proteins in HA-OGT and Flag-BRD4-transfected 293T cells and cardiomyocytes, respectively. In brief, the cells were lysed with the RIPA Lysis Buffer containing protease inhibitor cocktail. Subsequently, the cell lysates were incubated with the protein A/G beads at 4 °C for 4 h, followed by immunoprecipitation with anti-HA (ab236632, Abcam, UK), anti-OGT (ab96718, Abcam), or anti-IgG antibody at 4 °C overnight. The immunoprecipitated proteins were washed with IP lysis buffer and subjected to Western blotting.

### Detection of O-GlcNAc level of BRD4 protein

The O-GlcNAc levels of BRD4 protein were evaluated through immunoprecipitation of H9C2 and AC16 cell lysates using anti-HA (ab236632, Abcam, UK) or anti-BRD4 (M00123, Boster) at 4 °C overnight after protein A/G beads pre-incubation. The protein A/G beads were washed with IP lysis solution before Western blotting was used to measure the protein concentrations of O-GlcNAc, BRD4 and HA.

### Statistical analysis

All data are presented as mean ± standard deviation (SD). The statistical analysis was conducted using GraphPad Prism 8.0 software. A Student's t-test or one-way analysis of variance (ANOVA) with Tukey's post hoc test was used for comparisons between two groups or among several groups. ANOVA for repeated measures was used to examine the outcomes between two groups at various time points. A p-value of less than 0.05 was deemed statistically significant.

## Supplementary Material

Supplementary figure and tables.

## Abbreviations

CME: coronary microembolization
SRSF1: serine-arginine-rich splicing factor 1
ENPP3: ectonucleotide pyrophosphatase/phosphodiesterase 3
O-GlcNAc: O-linked N-acetylglucosamine
BRD4: bromodomain-containing protein 4
PBS: phosphate buffer saline
LVEF: left ventricular ejection fraction
LVEDd: left ventricular end-diastolic diameter
LVFS: left ventricular fractional shortening
CO: cardiac output
HE: haematoxylin and eosin
HBFP: hematoxylin-basic fuchsin-picric acid
DAB: 3,3′-diaminobenzidine
OGD: oxygen-glucose deprivation
shSRSF1: short hairpin RNA targeting SRSF1
shNC: negative control shRNA
ELISA: enzyme-linked immunosorbent assay
cTnI: cardiac troponin I
TNF-α: tumor necrosis factor-α
IL-1β: interleukin-1β
IL-6: interleukin-6
RT-qPCR: real-time quantitative PCR
PI: propidium iodide
RIP: RNA immunoprecipitation
TRAP: tagged RNA affinity purification
MS2bs: MS2-binding sequences
Co-IP: Co-immunoprecipitation
SD: standard deviation
ANOVA: analysis of variance

## References

[1] Kleinbongard P, Heusch G (2022). A fresh look at coronary microembolization. Nat Rev Cardiol.

[2] Heusch G, Skyschally A, Kleinbongard P (2018). Coronary microembolization and microvascular dysfunction. Int J Cardiol.

[3] Chang C, Huang WZ, Cai RP, Mo LR, Wu Q, Su Q (2025). Research Progress of Regulatory Cell Death in Coronary Microembolization. Int J Med Sci.

[4] Li L, Zheng Y, Li K, Kong L, Wang X, Zhou B (2024). Advances in MicroRNA-Mediated Regulation of Cardiomyocyte Injury After Coronary Microembolization. Cardiovasc Innov Appl.

[5] Dai R, Ren Y, Lv X, Chang C, He S, Li Q (2023). MicroRNA-30e-3p reduces coronary microembolism-induced cardiomyocyte pyroptosis and inflammation by sequestering HDAC2 from the SMAD7 promoter. Am J Physiol Cell Physiol.

[6] Anczuków O, Akerman M, Cléry A, Wu J, Shen C, Shirole NH (2015). SRSF1-Regulated Alternative Splicing in Breast Cancer. Mol Cell.

[7] van Bergeijk P, Seneviratne U, Aparicio-Prat E, Stanton R, Hasson SA (2019). SRSF1 and PTBP1 Are trans-Acting Factors That Suppress the Formation of a CD33 Splicing Isoform Linked to Alzheimer's Disease Risk. Mol Cell Biol.

[8] Lin L, Wang L, Li A, Li Y, Gu X (2024). CircDiaph3 aggravates H/R-induced cardiomyocyte apoptosis and inflammation through miR-338-3p/SRSF1 axis. J Bioenerg Biomembr.

[9] Gan T, Liu W, Wang Y, Huang D, Hu J, Wang Y (2024). LncRNA MAAMT facilitates macrophage recruitment and proinflammatory activation and exacerbates autoimmune myocarditis through the SRSF1/NF-κB axis. Int J Biol Macromol.

[10] Korekane H, Park JY, Matsumoto A, Nakajima K, Takamatsu S, Ohtsubo K (2013). Identification of ectonucleotide pyrophosphatase/phosphodiesterase 3 (ENPP3) as a regulator of N-acetylglucosaminyltransferase GnT-IX (GnT-Vb). J Biol Chem.

[11] Dong H, Liu Z, Wen H (2022). Protein O-GlcNAcylation Regulates Innate Immune Cell Function. Front Immunol.

[12] Silva JF, Olivon VC, Mestriner FLAC, Zanotto CZ, Ferreira RG, Ferreira NS (2020). Acute Increase in O-GlcNAc Improves Survival in Mice With LPS-Induced Systemic Inflammatory Response Syndrome. Front Physiol.

[13] Huang B, Yang XD, Zhou MM, Ozato K, Chen LF (2009). Brd4 coactivates transcriptional activation of NF-kappaB via specific binding to acetylated RelA. Mol Cell Biol.

[14] Dong X, Shu L, Zhang J, Yang X, Cheng X, Zhao X (2023). Ogt-mediated O-GlcNAcylation inhibits astrocytes activation through modulating NF-κB signaling pathway. J Neuroinflammation.

[15] Matsumoto E, Akiyama K, Saito T, Matsumoto Y, Kobayashi KI, Inoue J (2020). AMP-activated protein kinase regulates alternative pre-mRNA splicing by phosphorylation of SRSF1. Biochem J.

[16] Jimi E, Fei H, Nakatomi C (2019). NF-κB Signaling Regulates Physiological and Pathological Chondrogenesis. Int J Mol Sci.

[17] Hajmirza A, Emadali A, Gauthier A, Casasnovas O, Gressin R, Callanan MB (2018). BET Family Protein BRD4: An Emerging Actor in NFκB Signaling in Inflammation and Cancer. Biomedicines.

[18] Xu Y, Lv X, Cai R, Ren Y, He S, Zhang W (2022). Possible implication of miR-142-3p in coronary microembolization induced myocardial injury via ATXN1L/HDAC3/NOL3 axis. J Mol Med (Berl).

[19] Cai R, Xu Y, Ren Y, He S, Zheng J, Kong B (2022). MicroRNA-136-5p protects cardiomyocytes from coronary microembolization through the inhibition of pyroptosis. Apoptosis.

[20] Katsuyama T, Moulton VR (2021). Splicing factor SRSF1 is indispensable for regulatory T cell homeostasis and function. Cell Rep.

[21] Hu CK, Huang WZ, He L, Chang C, Ren YL, Dai RX (2025). De-succinylation-induced accumulation of TRMT10C in the nucleus plays a detrimental role in coronary microembolization via its m1A modification function. Int J Biol Sci.

[22] Li S, Zhong S, Zeng K, Luo Y, Zhang F, Sun X (2010). Blockade of NF-kappaB by pyrrolidine dithiocarbamate attenuates myocardial inflammatory response and ventricular dysfunction following coronary microembolization induced by homologous microthrombi in rats. Basic Res Cardiol.

[23] Chen A, Chen Z, Xia Y, Lu D, Jia J, Hu K (2018). Proteomics Analysis of Myocardial Tissues in a Mouse Model of Coronary Microembolization. Front Physiol.

[24] Lai HC, Ho UY, James A, De Souza P, Roberts TL (2021). RNA metabolism and links to inflammatory regulation and disease. Cell Mol Life Sci.

[25] Aubol BE, Serrano P, Fattet L, Wüthrich K, Adams JA (2018). Molecular interactions connecting the function of the serine-arginine-rich protein SRSF1 to protein phosphatase 1. J Biol Chem.

[26] Feng J, Xu X, Fan X, Yi Q, Tang L (2021). BAF57/SMARCE1 Interacting with Splicing Factor SRSF1 Regulates Mechanical Stress-Induced Alternative Splicing of Cyclin D1. Genes (Basel).

[27] Qian W, Liu F (2014). Regulation of alternative splicing of tau exon 10. Neurosci Bull.

[28] Tsai SH, Takeda K (2016). Regulation of allergic inflammation by the ectoenzyme E-NPP3 (CD203c) on basophils and mast cells. Semin Immunopathol.

[29] Zhang P, Wang C, Li C, Wang J (2024). miR-34a-5p Predicts the Risk of Diabetic Neuropathic Pain and Mediates Neuroinflammation in Microglia via Targeting ENPP3. Immunol Invest.

[30] Zhu Y, Ni H, Chen Q, Qian H, Fang Y, Gao R (2023). Inhibition of BRD4 expression attenuates the inflammatory response and apoptosis by downregulating the HMGB-1/NF-κB signaling pathway following traumatic brain injury in rats. Neurosci Lett.

[31] Schuetze KB, Stratton MS, Bagchi RA, Hobby ARH, Felisbino MB, Rubino M (2025). BRD4 inhibition rewires cardiac macrophages toward a protective phenotype marked by low MHC class II expression. Am J Physiol Heart Circ Physiol.

[32] Fang M, Luo J, Zhu X, Wu Y, Li X (2022). BRD4 Silencing Protects Angiotensin II-Induced Cardiac Hypertrophy by Inhibiting TLR4/NF-κB and Activating Nrf2-HO-1 Pathways. Cardiol Res Pract.

[33] Ben Ahmed A, Lemaire Q, Scache J, Mariller C, Lefebvre T, Vercoutter-Edouart AS (2023). O-GlcNAc Dynamics: The Sweet Side of Protein Trafficking Regulation in Mammalian Cells. Cells.

[34] Deng W, Chen Y, Zhang J, Ling J, Xu Z, Zhu Z (2024). Mild therapeutic hypothermia upregulates the O-GlcNAcylation level of COX10 to alleviate mitochondrial damage induced by myocardial ischemia-reperfusion injury. J Transl Med.

[35] Ling Y, Yang X, Zhang X, Guan F, Qi X, Dong W (2022). Myocardium-specific Isca1 knockout causes iron metabolism disorder and myocardial oncosis in rat. Life Sci.

