# Supplementary material for: Divergent splicing factor SRSF1 signaling promotes inflammation post-CME: the SRSF1/ENPP3 axis acts via inhibition of BRD4 O-GlcNAcylation to enhance NF-κB activation and accelerate heart failure
Source: Theranostics. 2025 Jun 9;15(14):6839–56. doi: 10.7150/thno.115402 (PMC12203807; doi:10.7150/thno.115402)
Supplement: Supplementary file 1 — Supplementary figure and tables. [file thnov15p6839s1.pdf]

Supplementary Materials

1. Supplementary Figure and figure legends

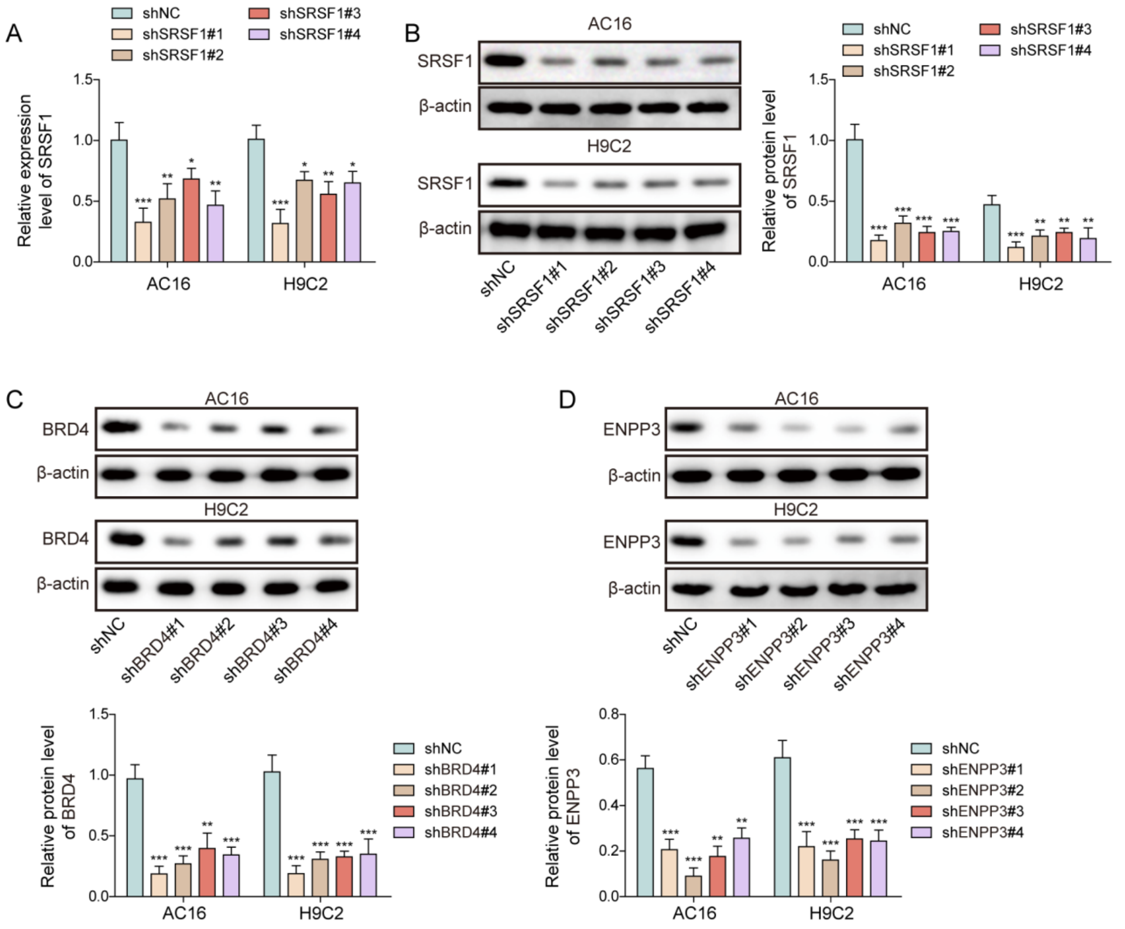

Figure S1 Silencing efficiency of shRNAs. (A)&(B) The silencing efficiency of shSRSF1#1, 2, 3, 4 was detected by RT-qPCR and Western blotting. (C) Western blot analysis of the silencing efficiency of shBRD4#1, 2, 3, 4. (D) The silencing efficiency of shENPP3#1, 2, 3, 4 was evaluated by Western blot. n=3 for A-D. one -way ANOVA was performed to analyze data. *$p < 0.05$, **$p < 0.01$, ***$p < 0.001$.

2. Supplementary table and table legends

Table S1. The shRNA sequences.

Gene	Name	shRNA targeting sequences
Human SRSF1	SH1	5′- GAGCTTTGATAGTCGTACCAT -3′
	SH2	5′-AGCTTTGATAGTCGTACCATA-3′
	SH3	5′- ACTGCCTACATCCGGGTTAAA-3′
	SH4	5′- ACTTACCTCCAGACATCCGAA -3′
Rat SRSF1	SH1	5′- GTGGAAGCTGGCAGGACTTAA -3′
	SH2	5′- GAAGCAGGTGATGTATGTTAT -3′
	SH3	5′- GTTTGTACGGAAAGAAGATAT -3′
	SH4	5′- ACTGCCTACATCCGGGTTAAA -3′
Human ENPP3	SH1	5′- CCAGACTTATTGTAACAAGAT -3′
	SH2	5′- GCACCAAACAATGGAACCCAT -3′
	SH3	5′- GCATGTAAAGACCGAGGTGAT -3′
	SH4	5′- CCGGATCAGAAGTGGCTATAA -3′
Rat SRSF1	SH1	5′- AGACAGCCTGAAGAGATATAA -3′
	SH2	5′- TGTGGACTCCATTCCAAATAT -3′
	SH3	5′- GTCAGTGCTGGAGTCATTAAA -3′
	SH4	5′- GCACATGGACCCAGCTTTAAA -3′

Human BRD4	SH1	5′- CCTGGAGATGACATAGTCTTA-3′
	SH2	5′- CGTCCGATTGATGTTCTCCAA -3′
	SH3	5′- CCTATGGATATGGGAACAATA -3′
	SH4	5′- GCCTATGTCCTATGAGGAGAA-3′
Rat BRD4	SH1	5′- CATGGATATGGGAACAATAAA -3′
	SH2	5′- GACTACTGTGACATCATTAAA -3′
	SH3	5′- GGCCGTGTAGTACACATAATT -3′
	SH4	5′- CAGACCAACCAACTGCAATAT -3′

Table S2. Primer sequences for RT-qPCR.

Gene	Forward (5’-3’)	Reverse (5’-3’)
**Human ENPP3**	GGGCTTGGACTCAGGAAACT	GAACAAGAGCAAAGGCTGGC
**Human lncRNA ENPP3**	ACAGTTTCTCTTTGCCAGACT	TCCTGGTAGCTGCATAGACCT
**Human ENPP3 pre-mRNA**	AGAGGGTGAATGAAAGTCCAAA	TGCTCCCATCAGCCAGAAT
**Human SRSF1**	TGCGTGAAGCAGGTGATGTA	GACCACTGTATCCAATTCTGGTCA
**Human TNF-α**	AGACCCTCACACTCAGATCATCTTC	CTCCGCTTGGTGGTTTGCTA
**Human IL-1β**	ATGATGGCTTATTACAGTGGCAA	GTCGGAGATTCGTAGCTGGA
**Human IL-6**	CCAGCTATGAACTCCTTCTC	GCTTGTTCCTCACATCTCTC
**Human BRD4**	GCACAATCAAGTCTAAACTGGAG	TCATGGTCAGGAGGGTTGTAC
**Human GAPDH**	GGCTGAGAACGGGAAGCTTGTCAT	CAGCCTTCTCCATGGTGGTGAAGA
**Rat ENPP3**	TGCCGAAACGACTGCACTAT	GTTGTAACCGTGTGTTCCGC
**Rat lncRNA ENPP3**	AAGAACAGCCGTCCACAATG	AAAGGAACTGTCCTGACTGCC
**Rat ENPP3 pre-mRNA**	TTCTACTGCTGCGAACTGACC	CCTACCTTGATGATGATGGACTG
**Rat SRSF1**	GTGGTTGTTTCTGGACTGCC	GGGCCCATCAACTTTAACCC
**Rat TNF-α**	ACCTTATCTACTCCCAGGTTCT	GGCTGACTTTCTCCTGGTATG
**Rat IL-1β**	AGACCTGACTTGGCAGAGA	GCAATGGTCGGGACATAGTT
**Rat IL-6**	GCCAGAGTCATTCAGAGCAATA	TTAGGAGAGCATTGGAAGTTGG

**Rat BRD4**	ACAACCCTCCTGACCATGAG	AACTGTCACTGTCCGAGGAG
**Rat GAPDH**	ACTCCCATTCTTCCACCTTTG	CCCTGTTGCTGTAGCCATATT
